# Machine learning based prediction of Williamson–Casson fluid flow with Cattaneo–Christov heat transfer over a curved stretching sheet

**DOI:** 10.1186/s11671-026-04789-y

**Published:** 2026-07-15

**Authors:** Ebrahem A. Algehyne, I. A. A. Manahill, Mohammed Rabih, Fahad Maqbul Alamrani, Anwar Saeed, Gabriella Bognár

**Affiliations:** 1 Department of Mathematics, Faculty of Science, University of Tabuk, P.O. Box 741, 71491 Tabuk, Saudi Arabia; 2 Computer Sciences Program, Department of Mathematics, Turabah University College, Taif University, P.O. Box 11099, 21944 Taif, Saudi Arabia; 3 Department of Mathematics, College of Science, Qassim University, 51452 Buraydah, Saudi Arabia; 4 Department of Computer Engineering, Biruni University, Istanbul, 34010 Turkey; 5 Institute of Machine and Product Design, University of Miskolc, Miskolc-Egyetemváros, 3515 Hungary

**Keywords:** Williamson–Casson fluid, Cattaneo–Christov heat flux, Curved stretching sheet, ANN, Melting heat condition, Magnetic dipole

## Abstract

Accurate prediction of non-Newtonian nanofluid flow and heat transfer under melting heat conditions is essential for applications in advanced thermal management systems, polymer extrusion and coating processes, metallurgical melting operations, biomedical fluid transport, micro- and nano-scale heat exchangers, and energy systems. Motivated by these practical applications, the current work systematically examines the Williamson-Casson nanofluid flow over a slippery curved expanding surface while simultaneously incorporating the Cattaneo-Christov heat flux model. The flow is affected by melting heat transfer conditions at boundary, activation energy, chemical reactivity and Soret/Dufour effects. The modeled equations have been solved through the bvp4c approach in dimensionless form. The solution obtained from this approach is then used to provide a dataset for Artificial Neural Network approach. It has discovered in this work that velocity profiles are augmented with growth in thermal buoyancy factor and velocity slip parameter, while declining with augmentation in magnetic parameter, ferrohydrodynamic interaction and Weissenberg number. Thermal profiles escalated with augmentation in heat source/sink parameter, heat dissipation factor, unsteadiness factor, radiation factor and Dufour number, while declining with augmentation in melting heat parameter, thermal buoyancy factor and thermal relaxation factor. The analysis of the histograms across the entire dataset by plotting the frequency of errors within discrete intervals confirms the validity and reliability of the model. With growth in local Weissenberg number, Casson factor and curvature parameter there is augmentation in skin friction. For higher Prandtl number and melting heat parameter there is intensification in Nusselt number. With escalation in Schmidt number and Soret number there is augmentation in Sherwood number while higher factor of activation energy causes reduction in it.

**Graphical abstract:**

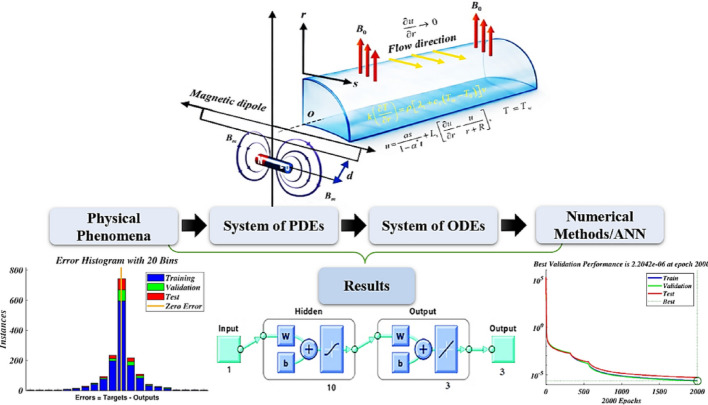

## Introduction

The study of Williamson-Casson fluid flow is an evolving area within non-Newtonian fluid mechanics, driven by the need to model fluids with complex rheological behavior. This combined model merges the characteristics of two distinct non-Newtonian fluids: the Casson fluid, which exhibits yield stress and shear-thinning properties (making it suitable for modeling substances like blood, tomato sauce, and molten chocolate), and the Williamson fluid, a pseudo-plastic model that describes fluids whose viscosity decreases with an increasing shear rate. Saleem et al. [1] examined Darcy-Forchheimer surface effects on magnetized Williamson-Casson fluid flow across an exponential extending surface. Paul and Nath [2] analyzed magneto-hydrodynamic Casson-Williamson fluid flow over an exponentially extending cylinder. Their study incorporated dissipation and injection/suction effects, examining how magnetic and non-Newtonian parameters influence velocity and temperature profiles to understand heat transfer characteristics in this complex boundary layer flow configuration [3]. Researchers have found that synthesizing these models into a Williamson-Casson fluid provides a more comprehensive framework for characterizing fluids useful in industrial and biomedical applications [4]. Al-Arabi et al. [5] investigated the magnetized flow of a Casson-Williamson fluid over a permeable sheet. Their study incorporated Darcy-Forchheimer porous medium effects, analyzing how these parameters influence the flow profiles of fluid. This combined fluid model has demonstrated practical relevance in emerging technologies, including wastewater treatment, solar energy systems, and microbial fuel cells, where understanding the complex flow dynamics is crucial for optimizing efficiency and performance as examined by Reddy et al. [6] and Abdelfattah et al. [7]. Supriya et al. [8] numerically studied time-dependent Casson-Williamson nanofluid flow over a vertical radiated cone. Hussain et al. [9] and Shoaib et al. [10] conducted a comprehensive numerical of the Casson nanofluid's flow under the magnetohydrodynamic (MHD) impact. The results demonstrate that the rising effect of magnetic field increases surface drag while suppressing convective heat transmission. Ahmad et al. [11] numerically assessed the thermal transport in unsteady micropolar hybrid nanoliquid flow consist of CeO_2_ and Al_2_O_3_ nanoparticles flow over a Riga plate.

The study of fluid flow incorporating the Cattaneo-Christov heat flux model represents a substantial progression in thermal transport theory, addressing the inherent limitations of the classical Fourier's law of heat conduction and Fick’s law for mass transference [12, 13]. While Fourier's law, which assumes a prompt thermal response to thermal gradients, remains widely used for its mathematical simplicity, it implies an unrealistic infinite speed of heat propagation. To resolve this paradox, the Cattaneo-Christov model [14, 15] familiarizes a thermal relaxation time parameter, accounting for the time lag mandatory to establish heat flow after a temperature gradient is imposed. Lone et al. [16] investigated magnetized nanofluid flow between two stretchable angular spinning plates. Utilizing Cattaneo-Christov flux theory with varying porosity, they analyzed how thermal relaxation and magnetic parameters influence velocity and temperature distributions in this complex rotating system. Sarma and Paul [17] discussed Darcy-Forchheimer fluid flow on a stretchable cylinder using the effects of Cattaneo-Christov flux theory. Contemporary research extensively applies this model to various non-Newtonian and Newtonian fluid flows to understand heat transfer under complex physical conditions. For instance, studies have examined its effects on magnetohydrodynamic (MHD) flows over stretching sheets, through porous media, and within boundary layer flows. A key finding across these investigations is that increasing the thermal relaxation parameter consistently leads to a drop in thermal distribution [18]. The Cattaneo-Christov framework has proven particularly valuable in engineering applications involving high-temperature processes, polymer extrusion, and nuclear reactor cooling, where accurate prediction of thermal behavior is critical [19]. By capturing non-Fourier effects, this model delivers an accurate description of heat transfer in materials with microscopic structure or those subjected to rapid thermal processing, enabling improved design and optimization in modern thermal systems. Abbas et al. [20] and Okasha et al. [21] examined the effects of Stefan blowing and heat generation on the Darcy–Forchheimer Ellis trihybrid nanoliquid flow across a sheet exposed to Cattaneo–Christov mass and heat flux using two different thermal conductivity models, the Yamada–Ota and the Xue models. Khan et al. [22] numerically estimated the Navier–Stokes and Maxwell equations for the fluid flow past a curved surface with the influence of buoyancy forces.

The study of fluid flow on a curved stretching sheet has emerged as a critically important area in fluid mechanics due to its direct relevance to numerous industrial and engineering processes. Unlike a flat stretching surface, a curved geometry introduces additional complexities arising from centrifugal forces and curvature-dependent pressure gradients, which fundamentally alter the boundary layer characteristics. When a sheet is stretched in a curved path, the flow is no longer unidirectional; the curvature generates a radial pressure variation that influences velocity profiles both inside and outside the boundary layer as noted by Saleem et al. [23] and Jan et al. [24]. Iqbal and Abbasi [25] studied magnetized fluid flow over a curved extendable surface by incorporating radial convection. Researchers have extensively investigated this configuration for various fluid types, including Newtonian, non-Newtonian, and nanofluids, under diverse physical conditions [26, 27]. A consistent finding across these studies is that increasing the curvature parameter significantly modifies the flow field. For smaller radii of curvature (higher curvature), the velocity near the sheet surface tends to decrease, while the associated temperature distribution often increases due to enhanced fluid friction and reduced momentum diffusion. Conversely, larger curvature radii produce behavior approaching that of a flat stretching sheet as examined also by Jan et al. [28]. Amajd et al. [29] highlighted that the curved stretching sheet geometry holds particular significance in polymer processing, where materials are often formed into curved shapes, in biomedical devices involving curved conduits, and in the cooling of curved electronic components. These flow dynamics enables engineers to predict skin friction coefficients and Nusselt numbers accurately, facilitating optimized design of thermal systems where curved surfaces undergo stretching during manufacturing or operation. Lu et al. [30] investigated nonlinear thermal radiation effects on nanofluid flow over a curved elongating surface. Their analysis revealed how radiation parameters influence temperature distribution and heat transfer characteristics, demonstrating enhanced thermal performance with increasing nonlinear radiation in this curved geometry configuration. Some recent results on curved and stretching surfaces are reported by Ref. [31–34].

The integration of Artificial Neural Networks (ANNs) into the study of fluid flow symbolizes a transformative paradigm shift in computational fluid dynamics, offering powerful data-driven alternatives to traditional numerical and analytical methods. Lone et al. [35] deliberated on the production entropy for stagnant fluid flow on a Riga surface with nonlinear convection using ANN approach. In fluid mechanics research, ANNs are increasingly employed to predict velocity profiles, across a wide range of flow configurations, including boundary layer flows over stretching surfaces, magnetohydrodynamic (MHD) flows, nanofluid transport, and flows through porous media [36–38]. Bilal et al. [39] scrutinized stagnation point nanofluid flow over a Riga surface with nonlinear thermal convection. Using the ANN technique, they examined how modified Hartmann number and convection parameters influence velocity and temperature distributions in this magnetohydrodynamic configuration. As computational capabilities continue to advance and data availability increases, ANN-based methods are poised to become indispensable tools in modern fluid dynamics research, enabling faster predictions, uncertainty quantification, and the discovery of hidden relationships within complex flow phenomena. The ANN approach proves particularly valuable when dealing with highly non-linear coupled differential equations that challenge traditional solvers or when experimental data is available but theoretical models are incomplete [40]. Furthermore, hybrid approaches combining ANNs with optimization algorithms enable inverse modeling, allowing researchers to determine optimal flow parameters for desired thermal or hydrodynamic outcomes. Recent studies have demonstrated the exceptional capability of ANNs in modeling complex non-Newtonian fluid phenomena, including Casson-Williamson and Maxwell fluids etc. [41]. The typical methodology involves generating training data through established numerical techniques followed by training the ANN model using algorithms to minimize prediction errors. Once trained, these networks can instantly predict flow behavior for new parameter combinations with remarkable accuracy [42].

The study of fluid flow incorporating melting heat transfer conditions represents a fascinating and practically substantial area of thermal fluid science, with insightful implications across multiple engineering disciplines. Melting heat transfer occurs when a flowing fluid interacts with a solid interface undergoing phase change from solid to liquid, absorbing latent heat at the boundary and fundamentally altering the thermal and flow characteristics of the system. This phenomenon is mathematically modeled through a specialized boundary condition that balances conductive heat transfer into the solid with the latent heat required for melting, typically expressed as the melting parameter that relates the solid's latent heat to the fluid's sensible heat. Prakash and Tripathi [43] examined stagnant point fluid flow on stretching surface with melting heat transference constraints at boundary. When a melting surface is present, the no-slip condition at the boundary is modified as liquid from the melted solid is continuously introduced into the flow field, affecting momentum transport and boundary layer development as examined by Khan et al. [44] and Feng et al. [45]. Contemporary research has extensively examined melting heat transfer across diverse fluid systems, including Newtonian and non-Newtonian fluids, nanofluids, and magnetohydrodynamic (MHD) flows over various geometries. Mahariq et al. [46] highlighted that for such flow increasing the melting parameter significantly reduces both temperature and velocity distributions. This occurs because the phase change process absorbs substantial thermal energy, effectively cooling the near-wall region and diminishing temperature gradients, which consequently affects fluid viscosity and momentum transport. Melting heat transfer enables engineers to optimize industrial processes involving phase change [47], design more efficient thermal management systems, and develop accurate models for natural phenomena where solid–liquid phase transitions occur [48]. Ullah et al. [49] investigated magnetized radiative fluid flow over an extendable surface incorporating melting, thermal generation, activation energy, and velocity slippage. Their analysis revealed how these coupled phenomena influence thermal and momentum transport characteristics significantly. Furthermore, melting heat transfer intensely influences engineering applications ranging from semiconductor crystal growth and casting processes to thermal energy storage systems using phase change materials [50]. In geophysical contexts, it governs magma dynamics and ice sheet melting, while in biomedical engineering, it relates to cryopreservation and laser-tissue interactions.

### Novelty

Based on a critical evaluation of the existing literature, it is evident that no prior study has systematically examined the Williamson-Casson nanofluid flow over a slippery curved expanding surface while simultaneously incorporating the Cattaneo-Christov heat flux model, cross diffusion and melting heat transfer conditions, particularly through the application of machine learning algorithms. This absence highlights a clear and substantial research gap in literature. To address this deficiency, the present study undertakes a comprehensive investigation of the coupled nonlinear transport phenomena governing such fluid behavior by various effects. The flow is affected by melting heat transfer conditions at the boundary, activation energy, chemical reactivity and Soret/Dufour effects. The modelled equations have been solved through the bvp4c approach in dimensionless form. The solution obtained from this approach is then used to provide a dataset for the Artificial Neural Network (ANN) approach.

## Problem formulation

**Fig. 1:**
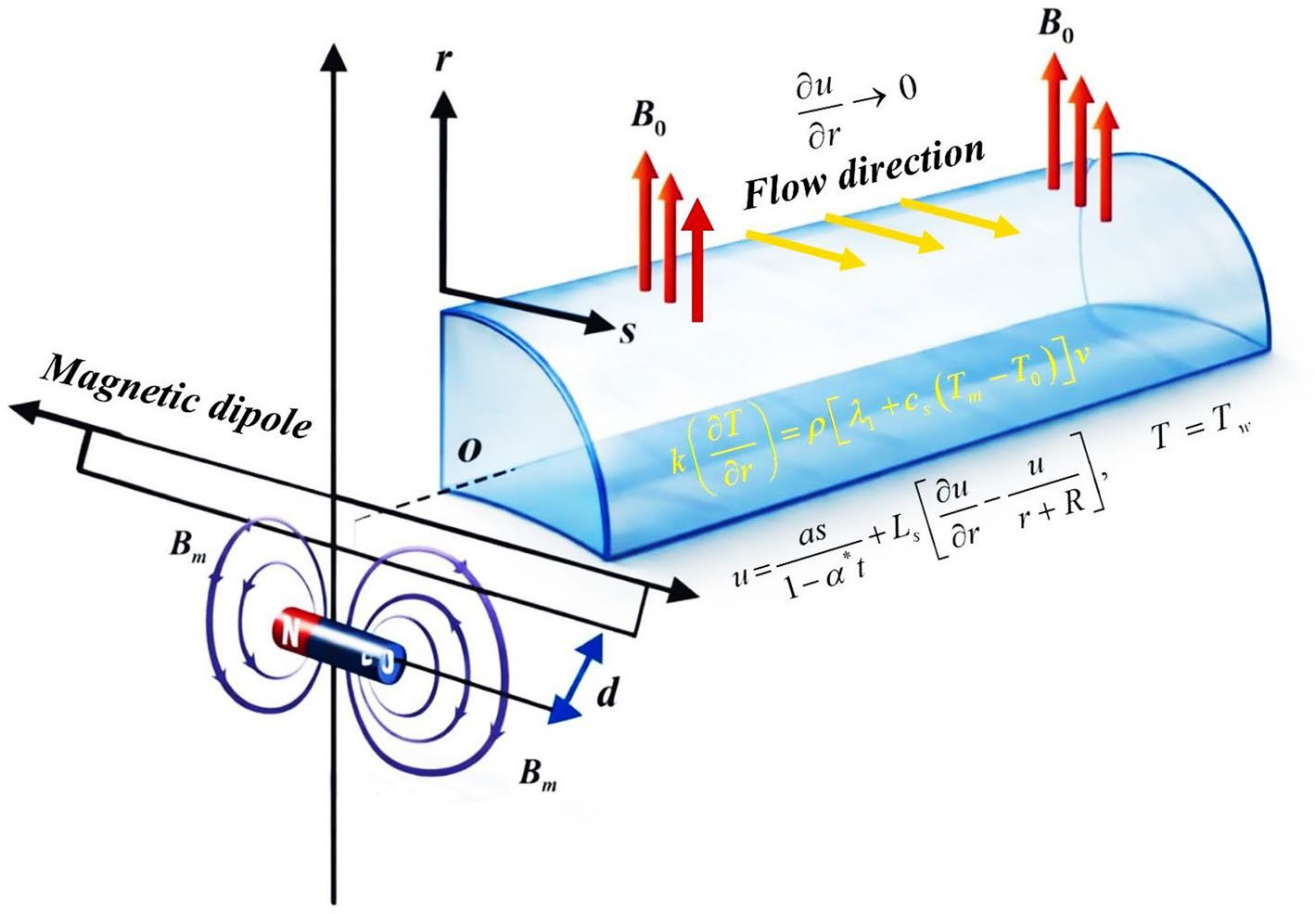
Flow mechanism subject to the magnetic dipole

Consider the unsteady flow of Williamson-Casson (WC) fluid across a curved stretching surface. The surface is stretching about an arc with radius *R* subject to two equal and opposite surface pressures, whereas the radial direction *r* is normal to it (Fig. 1). The stretching rate of the sheet is described as $u_{w} \left( s \right) = \frac{as}{{1 - \alpha^{*} t}}$, where *t*, *a* and $\alpha^{*}$ is the time, elongating rate, and a constant reciprocal to the dimension of time. The effect of the magnetic dipole $B_{m} = \frac{{B_{0} }}{{\sqrt {1 - \alpha^{*} t} }}$ is employed towards *r*-axis. The influence of buoyancy is examined to analyze the flow properties of the nanofluid. The energy equation accounts for the effects of Joule heating and heat radiation to analyze the mechanics of the flow. The impacts of the CCHF model on flow have been thoroughly examined.

Using the above stated assumptions the flow equations are defined as [51, 52]:

1 $$ \frac{\partial }{\partial r}\left( {\left( {R + r} \right)v} \right) + R\frac{\partial u}{{\partial s}} = 0, $$

2 $$ \rho \frac{{u^{2} }}{r + R} = \frac{\partial p}{{\partial r}}, $$

3 $$ \begin{gathered} \frac{\partial u}{{\partial t}} + v\frac{\partial u}{{\partial r}} + \frac{R}{r + R}u\frac{\partial u}{{\partial s}} + \frac{uv}{{r + R}} = - \frac{R}{{\rho \left( {r + R} \right)}}\frac{\partial p}{{\partial s}} + \upsilon \left[ \begin{gathered} \sqrt {2 \Gamma } \left\{ \begin{gathered} \frac{\partial u}{{\partial r}} \frac{{\partial^{2u} }}{{\partial r^{2} }} + \frac{1}{r + R}\left\{ {\left( {\frac{\partial u}{{\partial r}}} \right)^{2} - u\frac{{\partial^{2u} }}{{\partial r^{2} }}} \right\} + \frac{{u^{2} }}{{\left( {r + R} \right)^{3} }} - \hfill \\ \frac{2u}{{\left( {r + R} \right)^{2} }}\frac{\partial u}{{\partial r}} \hfill \\ \end{gathered} \right\} \hfill \\ \left( {1 + \frac{1}{\beta }} \right)\left( {\frac{{\partial^{2u} }}{{\partial r^{2} }} + \frac{1}{r + R}\frac{\partial u}{{\partial r}} - \frac{u}{{\left( {r + R} \right)^{2} }}} \right) \hfill \\ \end{gathered} \right] \hfill \\ + \frac{{\mu_{0} M}}{\rho }\frac{\partial H}{{\partial s}} + g\left( {\beta_{T} \left( {T - T_{\infty } } \right)} \right) - \frac{\sigma }{\rho }B_{m}^{2} u, \hfill \\ \end{gathered} $$

4 $$ \begin{gathered} \frac{\partial T}{{\partial t}} + v\frac{\partial T}{{\partial r}} + R\frac{u}{r + R}\frac{\partial T}{{\partial s}} = \alpha \left( {\frac{{\partial^{2} T}}{{\partial r^{2} }} + \frac{1}{r + R}\frac{\partial T}{{\partial r}}} \right) + \tau \left[ {D_{m} \left( {\frac{\partial C}{{\partial r}}\frac{\partial T}{{\partial r}}} \right) + \frac{{D_{T} }}{{T_{\infty } }}\left( {\frac{\partial T}{{\partial r}}} \right)^{2} } \right] - \frac{1}{{\rho c_{p} }}\frac{1}{r + R}\frac{{\partial \left[ {\left( {r + R} \right)q_{r} } \right]}}{\partial r} - \lambda_{1} \hfill \\ \left[ {v^{2} \frac{{\partial^{2} T}}{{\partial r^{2} }} + u^{2} \left( {\frac{R}{r + R}} \right)^{2} \frac{{\partial^{2} T}}{{\partial s^{2} }} + \left( {v\frac{\partial v}{{\partial r}} + \frac{uR}{{r + R}}\frac{\partial v}{{\partial s}}} \right)\frac{\partial T}{{\partial s}} + \left( {\frac{vR}{{r + R}}\frac{\partial u}{{\partial r}} + u\left( {\frac{R}{r + R}} \right)^{2} \frac{\partial u}{{\partial s}}} \right)\frac{\partial T}{{\partial s}} + \frac{2uvR}{{r + R}}\frac{{\partial^{2} T}}{\partial r\partial s}} \right] + \frac{{Q_{0} }}{{\rho C_{p} }} \hfill \\ \left( {T - T_{\infty } } \right)e^{{\left( { - \sqrt {\frac{a}{\nu }} r} \right)}} + \frac{1}{{\rho C_{p} }}\left[ {u\frac{\partial H}{{\partial s}} + \frac{\partial H}{{\partial r}}} \right]\mu_{0} T\frac{\partial M}{{\partial T}} + \left( {\frac{\sigma }{{\rho C_{p} }}} \right)B_{m}^{2} u^{2} + \frac{{D_{m} k_{T} }}{{c_{s} }}\left( {\frac{{\partial^{2} C}}{{\partial r^{2} }} + \frac{1}{r + R}\frac{\partial C}{{\partial r}}} \right), \hfill \\ \end{gathered} $$

5 $$ \begin{aligned} \left( {\frac{{\partial T}}{{\partial t}} + v\frac{{\partial C}}{{\partial r}} + \left( {\frac{R}{{r + R}}} \right)u\frac{{\partial C}}{{\partial s}}} \right) = & \left( {\frac{{\partial ^{2} C}}{{\partial r^{2} }} + \left( {\frac{1}{{r + R}}} \right)\frac{{\partial C}}{{\partial r}}} \right)D_{m} - \frac{{k_{r}^{2} }}{{C_{\infty } }}\left( {\frac{T}{{T_{\infty } }}} \right)^{n} e^{{ - \frac{{Ea}}{{K_{1} T}}}} \left( {C - C_{\infty } } \right) \\ & \quad + \left( {\frac{{\partial ^{2} T}}{{\partial r^{2} }} + \frac{1}{{r + R}}\frac{{\partial T}}{{\partial r}}} \right)\frac{{D_{m} c_{s} k_{T} }}{{T_{m} }}. \\ \end{aligned} $$

The IBCs (initial and boundary conditions) are [51, 52]:

6 $$ \begin{gathered} u = \frac{as}{{1 - \alpha^{*} t}} + L_{s} \left[ {\frac{\partial u}{{\partial r}} - \frac{u}{r + R}} \right], k\left( {\frac{\partial T}{{\partial r}}} \right) = \rho \left[ {\lambda_{1} + c_{s} \left( {T_{m} - T_{0} } \right)} \right]v, \,\,T = T_{w} ,\,\,C = C_{w} , \,\,{\mathrm{at}}\,\, r = 0, \hfill \\ u \to 0, \,\,\,v \to 0,\,\,\,\frac{\partial u}{{\partial r}} \to 0, \,\,\,T \to T_{\infty } , \,\,\,C \to C_{\infty } \,\,{\mathrm{as}}\,\, r \to \infty . \hfill \\ \end{gathered} $$

Here, $\left( {u,v} \right)$ are the velocity component towards $\left( {s,r} \right)$, $\upsilon$ and $\rho$ are the kinematic viscosity and density,$p$ is the pressure, $\beta$ and ${\Gamma }$ are the Casson factor and constant of material time, $g$ and $\beta_{T}$ are the acceleration and coefficient of thermal expansion, $\mu_{0}$ and $M$ are the magnetic permeability and magnetization, $H$ and $\sigma$ are the magnetic field and electrical conductivity,$\beta_{0}$ and $T$ are the magnetic field and temperature, $Q_{0}$ and $C_{p}$ are the heat source and specific heat, $\alpha$ and *τ* are the thermal diffusivity and ratio of effective heat capacity,$q_{r}$ and $\lambda_{1}$ are the radiative heat flux and thermal relaxation time, $D_{T}$ and $T_{w}$ are the factor of thermophoretic diffusion and surface temperature, $\lambda_{l}$ and $T_{\infty }$ are the fluid latent heat and ambient temperature of fluid, ($k_{c}$, $k_{s}$) and $L_{s}$ are the constant for reaction rate and slip velocity, $C_{s}$ and $T_{m}$ are the heat capacity of solid surface and the melting temperature, $T_{0}$ and *k* are the solid surface temperature and thermal conductivity.

According to the Rosseland approximation, the radiative heat flow can be described as:

7 $$ q_{r} = - \frac{{4 \sigma^{*} }}{{3 k^{*} }}\left( {\frac{{\partial T^{4} }}{\partial r}} \right) = - \frac{{16 \sigma^{*} T_{\infty }^{3} }}{{3 k^{*} }}\left( {\frac{\partial T}{{\partial r}}} \right). $$

*Magnetic dipole*. The influence of the magnetic field on the liquid stream is dictated by the observable magnetic dipole and its associated scalar strength.

8 $$ \Phi = \frac{\gamma }{2\pi }\left\{ {\frac{s}{{ s^{2} + \left( {r + d} \right)^{2} }}} \right\}. $$

Here, *d* represents the distance between the dipoles, and $\gamma$ denotes the magnetic field strength. The characteristics associated with the magnetic field *H* are as follows:

9 $$ H_{r} = - \frac{\partial \Phi }{{\partial r}} = \frac{\gamma }{2\pi }\frac{{2s\left( {r + d} \right)}}{{\left\{ {s^{2} + \left( {r + d} \right)^{2} } \right\}^{2} }},\,\,\, H_{s} = - \frac{\partial \Phi }{{\partial s}} = \frac{\gamma }{2\pi }\frac{{s^{2} - \left( {r + d} \right)}}{{\left\{ {s^{2} + \left( {r + d} \right)^{2} } \right\}^{2} }}. $$

The subsequent correlation indicates that the amount of H varies directly with the temperature and magnetic force, as estimated linearly from M, characterized as:

10 $$ H = \sqrt {H_{r}^{2} + H_{s}^{2} } ,\,\,\,M = K_{1} \left( {T - T_{\infty } } \right). $$

The similarity variables are:

11 $$ \left. \begin{gathered} p = \frac{{\rho a^{2} s^{2} }}{{\left( {1 - \alpha^{*} t} \right)^{2} }}P\left( \eta \right),\,\,\,v = \frac{ - R}{{r + R}}\sqrt {\frac{av}{{1 - \alpha^{*} t}}} f\left( \eta \right), u = \frac{as}{{1 - \alpha^{*} t}}f^{\prime}\left( \eta \right),\,\,\,\,\varphi = \frac{{C - C_{\infty } }}{{C_{w} - C_{\infty } }},\,\,\,\,\kappa = \sqrt {\frac{a}{{\nu \left( {1 - \alpha^{*} t} \right)}}} R, \hfill \\ \eta = \sqrt {\frac{a}{{\nu \left( {1 - \alpha^{*} t} \right)}}} r,\,\,\,\,\theta = \frac{{T - T_{\infty } }}{{T_{w} - T_{\infty } }}. \hfill \\ \end{gathered} \right\} $$

Here, $K_{1}$ is the ferromagnetic coefficient, $\kappa$ and $\eta$ are the curvature factor and similarity variable. By using Eq. (11) in modeled equations, we get:

12 $$ \frac{dP}{{d\eta }} = \frac{{f^{{\prime}{2}} }}{\eta + \kappa }, $$

13 $$ \left. \begin{gathered} \left( {\frac{2\kappa }{{\eta + \kappa }}} \right)P = \frac{\kappa }{{\left( {\eta + \kappa } \right)^{2} }}ff^{\prime} + \frac{\kappa }{\eta + \kappa }f f^{\prime\prime} - \frac{\kappa }{\eta + \kappa }f^{{\prime}{2}} + \left( {1 + \frac{1}{\beta }} \right)\left[ {f^{\prime\prime\prime} + \frac{1}{{\left( {\eta + \kappa } \right)}}f^{\prime\prime} - \left( {\frac{1}{{\left( {\eta + \kappa } \right)^{2} }}} \right)f^{\prime}} \right] + We \hfill \\ \left( { f^{\prime\prime}f^{\prime\prime\prime} + \frac{1}{{\left( {\eta + \kappa } \right)}}\left( {f^{\prime \prime 2} - f^{\prime}f^{\prime\prime\prime}} \right) - \frac{{2f^{\prime}f^{\prime\prime}}}{{\left( {\eta + \kappa } \right)^{2} }} + \frac{{f^{{\prime}{2}} }}{{\left( {\eta + \kappa } \right)^{3} }}} \right) - \delta^{*} \left( { f^{\prime} + \frac{\eta }{2}f^{\prime\prime}} \right) + \lambda_{T} \theta - M^{*} f^{\prime} - \frac{{2\beta_{m} }}{{\left( {n + b} \right)^{4} }}\theta , \hfill \\ \end{gathered} \right\} $$

14 $$ \left. \begin{gathered} \frac{1}{Pr}\left[ {1 + Rd} \right]\left( { \theta^{\prime\prime} + \left( {\frac{1}{\eta + \kappa }} \right)\theta^{\prime}} \right) + \frac{\kappa }{\eta + \kappa }f \theta^{\prime} + Nb \varphi^{\prime}\theta^{\prime} + Nt \left( {\theta^{\prime}} \right)^{2} - \left( {\frac{\eta }{2}} \right)\delta^{*} \theta^{\prime} + Q \theta e^{ - \eta } + Ec M^{*} \left( {f^{\prime}} \right)^{2} + D_{f} \hfill \\ \left( {\varphi^{\prime\prime} + \frac{1}{\eta + K}\varphi^{\prime}} \right) - C_{H} \left( {\frac{\kappa }{\eta + \kappa }} \right)^{2} \left[ \begin{gathered} f^{2} \theta^{\prime\prime} + f f^{\prime}\theta^{\prime} \hfill \\ - \left( {\frac{{f^{2} }}{\eta + \kappa }} \right)\theta^{\prime} \hfill \\ \end{gathered} \right] + \frac{2}{Pr}\frac{{\beta_{m} \lambda_{m} \left( {\theta - \varepsilon } \right)}}{{\left( {\eta + b} \right)^{3} }}\left[ {\frac{Kf}{{\left( {\eta + \kappa } \right)}}\left\{ {1 - \frac{2}{{\left( {\eta + b} \right)^{2} }}} \right\} - \frac{{f^{\prime}}}{\eta + b}} \right] = 0, \hfill \\ \end{gathered} \right\} $$

15 $$ \varphi^{\prime\prime} + \frac{1}{\eta + K}\varphi^{\prime} + Sc\left( {\frac{K}{\eta + K}f\varphi^{\prime} - K_{r} \left( {1 + \omega \theta } \right)^{n} Exp\left( { - \frac{{E_{1} }}{1 + \omega \theta }} \right) - \frac{\eta }{2}\delta^{*} \varphi^{\prime} + \left( {\theta^{\prime\prime} + \frac{1}{\eta + K}\theta^{\prime}} \right)Sr} \right) = 0. $$

The transform IBCs are:

16 $$ \left. \begin{gathered} f^{\prime} = 1 + L_{1} \left( { f^{\prime\prime} - \left( {\frac{1}{\kappa }} \right)f^{\prime}} \right), M_{e} \theta^{\prime} + Prf = 0, \theta = 1, \varphi = 1 \,\,{\mathrm{at}} \,\,\eta = 0 \hfill \\ f^{\prime} \to 0, \,\,f^{\prime\prime} \to 0, \,\,\theta \to 0,\,\,\, \varphi \to 1 \,\,\,{\mathrm{as}}\,\,\, \eta \to \infty . \hfill \\ \end{gathered} \right\} $$

In above equations, $We = \sqrt {\frac{{2a^{3} }}{{\nu \left( {1 - \alpha^{*} t} \right)^{3} }}} {\Gamma }s$ is local Weissenberg number, $\delta^{*} = \frac{{\alpha^{*} }}{a}$ is unsteadiness parameter, $\beta_{m} = \frac{{\gamma \mu_{0} K_{1} \left( {T_{w} {-} T_{\infty } } \right)\rho }}{{2\pi \mu^{2} }}$ is ferrohydrodynamic interaction,$Sr = \frac{{D_{m} c_{s} k_{T} \left( {T_{w} - T_{\infty } } \right)}}{{T_{m} \nu \left( {C_{w} - C_{\infty } } \right)}}$ is Soret number, $b = \sqrt {\frac{a}{{\nu \left( {1 - \alpha^{*} t} \right)}}} d$ is dimensionless distance, $Gr = \frac{{g \beta_{T} T_{\infty } \left( {\theta_{w} {-} 1} \right)s^{3} }}{{\nu^{2} }}$ is local Grashof number, $K_{r} = \frac{{k_{r}^{2} }}{{C_{\infty } b}}$ is rate of chemical reaction, $\lambda_{T} = \frac{Gr}{{Re^{2} }}$ is thermal buoyancy parameter, $M^{*} = \frac{{\sigma B_{0}^{2} }}{\rho a}$ is magnetic parameter, $Rd = \frac{{16 \sigma^{*} T_{\infty }^{3} }}{{3 k k^{*} }}$ is thermal radiation parameter,$D_{f} = \frac{{D_{m} k_{T} \left( {C_{w} - C_{\infty } } \right)}}{{c_{s} \left( {\mu C_{p} } \right)\left( {T_{w} - T_{\infty } } \right)}}$, $Pr = \frac{\upsilon }{\alpha }$ is Prandtl number,$E_{1} = \frac{Ea}{{K_{1} T_{\infty } }}$ is factor of activation energy, $Nt = \frac{{\tau D_{T} \left( {T_{w} {-} T_{\infty } } \right)}}{{\nu T_{\infty } }}$ is thermophoresis parameter,$Sc = \frac{{\upsilon_{f} }}{{D_{m} }}$ is Schmidt number, $Nb = \frac{{\tau D_{m} C_{0} }}{\upsilon }$ is Brownian motion parameter, $C_{H} = \lambda_{1} a_{1}$ is thermal relaxation parameter, $Ec = \frac{{u_{w}^{2} }}{{C_{p} \left( {T_{w} {-} T_{\infty } } \right)}}$ is Eckert number, $\varepsilon = \frac{{T_{\infty } }}{{T_{\infty } - T_{w} }}$ is curie temperature, $\lambda_{m} = \frac{{a \mu^{2} }}{{\rho k \left( {T_{w} {-} T_{\infty } } \right)\left( {1 - \alpha^{*} t} \right)}}$ is heat dissipation parameter, $Q = \frac{{Q_{0} \left( {1 - \alpha^{*} t} \right)}}{{a \left( {\rho C_{p} } \right)}}$ is heat source/sink parameter, $M_{e} = \frac{{C_{p} \left( {T_{w} {-} T_{\infty } } \right)}}{{\lambda_{1} + \left( {T_{w} {-} T_{0} } \right)c_{s} }}$ is melting heat parameter, and $L_{1} = L_{s} \sqrt {\frac{a}{{\upsilon \left( {1 - \alpha^{*} t} \right)}}}$ is slip parameter.

By eliminating the pressure term from Eqs. (12) and (13):

17 $$ \begin{gathered} \left( {1 + \frac{1}{\beta }} \right)\left[ {f^{{\prime v}} + ~\frac{{2f^{\prime\prime\prime}}}{{\eta + \kappa }} - \frac{{f^{\prime\prime}}}{{\left( {\eta + \kappa } \right)^{2} }} + \frac{{f^{\prime}}}{{\left( {\eta + \kappa } \right)^{3} }}} \right]~~ + ~We\left[ \begin{gathered} \left( {f^{\prime\prime}\,f^{{\prime v}} + f^{\prime\prime\prime 2}} \right) - \frac{1}{{\left( {\eta + \kappa } \right)^{2} }}\left( {f^{\prime}\,f^{{\prime v}} - 2f^{\prime\prime}\,f^{\prime\prime\prime}} \right) - \frac{2}{{\left( {\eta + \kappa } \right)^{2} }} \hfill \\ \left( {f^{\prime\prime 2} + f^{\prime}\,f^{\prime\prime\prime}} \right) + \frac{{4f^{\prime}\,f^{\prime\prime}}}{{\left( {\eta + \kappa } \right)^{3} }} - \frac{{2f^{{\prime 2}} }}{{\left( {\eta + \kappa } \right)^{4} }} \hfill \\ \end{gathered} \right] \hfill \\ + ~\frac{\kappa }{{\eta + \kappa }}\left[ {f~f^{\prime\prime\prime} - ~f^{\prime}f^{\prime\prime}} \right] + ~\frac{\kappa }{{\left( {\eta + \kappa } \right)^{2} }}\left[ {ff^{\prime\prime} - ~f^{{\prime 2}} } \right] - ~\frac{\kappa }{{\left( {\eta + \kappa } \right)^{3} }}f~f^{\prime} - ~\delta ^{*} \left( {~\left( {\frac{\eta }{2}} \right)f^{\prime\prime\prime} + ~\frac{{3f^{\prime\prime}}}{2}} \right) - ~\frac{{\delta ^{*} }}{{\left( {\eta + \kappa } \right)}}\left[ {\left( {\frac{\eta }{2}} \right)f^{\prime\prime} + ~f^{\prime}} \right] \hfill \\ ~ - \frac{{2\beta _{m} }}{{\left( {\eta + b} \right)^{4} }}\left[ {~\left( {\frac{1}{{\eta + \kappa }} - \frac{4}{{\eta + b}}} \right)\theta ~ + ~\theta ^{\prime}} \right] + ~\lambda _{T} \left[ {\frac{\theta }{{\eta + \kappa }} + ~\theta ^{\prime}} \right] - ~M^{*} \left[ {f^{\prime\prime} + \frac{{f^{\prime}}}{{\eta + \kappa }}} \right] = ~0. \hfill \\ \end{gathered} $$

The Nusselt number and skin friction coefficient are:

18 $$ Nu_{s} = \frac{{sq_{w} }}{{\left( {T_{w} - T_{\infty } } \right)k}},\,\,\,\,Cf_{s} = \frac{{\tau_{w} }}{{\rho u_{w}^{2} }},\,\,\,Sh_{s} = \frac{{sj_{w} }}{{\left( {C_{w} - C_{\infty } } \right)D_{m} }}. $$

Here $q_{w} ,\,\,\tau_{w} \,\,{\mathrm{and}}\,\,j_{w}$ are the heat flux, wall shear stress and wall mass flux:

19 $$ \begin{gathered} q_{w} = \left. { - k \left( {\frac{\partial T}{{\partial r}}} \right)} \right|_{r = 0} + \left. {q_{r} } \right|_{r = 0} ,\,\,\,\tau_{w} = \left. {\mu \left[ {\left( {1 + \frac{1}{\beta }} \right)\left( {\frac{\partial u}{{\partial r}} + \frac{R}{R + r}\frac{\partial v}{{\partial s}} - \frac{u}{r + R}} \right) + \left( {\frac{\partial u}{{\partial r}} - \frac{u}{r + R} + \frac{R}{R + r}\frac{\partial v}{{\partial s}}} \right)^{2} \left( {\frac{{{\Gamma }^{2} }}{\sqrt 2 }} \right)} \right]} \right|_{r = 0} , \hfill \\ j_{w} = \left. {D_{m} \left( {\frac{\partial C}{{\partial r}}} \right)} \right|_{r = 0} . \hfill \\ \end{gathered} $$

By using Eq. (19) in Eq. (18), we get:

20 $$ \begin{gathered} Nu_{s} \frac{1}{{\sqrt {Re} }} = - \left( {1 + Rd} \right)\theta^{\prime}\left( 0 \right),\,\,Cf_{s} \sqrt {Re} = \left( {1 + \frac{1}{\beta }} \right) \left[ { f^{\prime\prime}\left( 0 \right) - \left( {\frac{1}{\kappa }} \right)f^{\prime}\left( 0 \right)} \right] + \left( \frac{We}{2} \right) \left[ {f^{\prime\prime}\left( 0 \right) - \left( {\frac{1}{\kappa }} \right)f^{\prime}\left( 0 \right)} \right]^{2} \hfill \\ Sh_{s} = \frac{1}{{\sqrt {Re} }} = - \varphi^{\prime}\left( 0 \right). \hfill \\ \end{gathered} $$

In Eq. (20), *Re* = $\frac{{as^{2} }}{\upsilon }$ is the Reynolds number.

## Numerical algorithm

To solve the system of differential equations presented in Eqs. (12–15) using Eq. (16), the renowned numerical solver bvp4c has been employed. This solver, integrated into the MATLAB environment, is a powerful and widely used tool for handling non-linear ODEs. Its application is particularly well-suited for this study due to its robust algorithm, which is based on the collocation method. The solver operates by dividing the domain of interest into a mesh of points and approximating the solution as a piecewise polynomial across each subinterval. A key advantage of bvp4c is its fast convergent technique, which allows it to efficiently refine the mesh and converge to an accurate solution even when dealing with severe nonlinearities, sharp gradients, or singularities that are common in advanced fluid mechanics and heat transfer problems.

For severe non-linearities, to assure a high level of computational stability and accuracy, certain solver parameters and error tolerances are applied in the bvp4c framework. The numerical calculations are performed with absolute (AbsTol) and relative (RelTol) tolerances of 10⁻⁶ and 10⁻⁷ respectively. An automated adaptive mesh selection procedure, based on the residual of the continuous piecewise polynomial solution, is used in the algorithm. It starts with the initial uniform-mesh of 100 points, but the maximum possible number of points is increased to 1000, dynamically, to resolve sharp velocity and thermal gradients near the curved stretching boundary (*η* = 0). The computational stability is verified by checking that the residual profiles are uniformly bounded below 10^–5^ over the whole domain for the grid, so that the data obtained for the initial grid is absolutely convergent and reliable and can be used for subsequent network training [53, 54].

To employ bvp4c, the system is first reduced into 1st order as:

21 $$ \varpi_{1} = f,\,\,\,\varpi_{2} = f^{\prime},\,\,\,\varpi_{3} = f^{\prime\prime},\,\,\,\varpi_{4} = f^{\prime\prime\prime},\,\,\,\varpi_{5} = \theta ,\,\,\,\varpi_{6} = \theta^{\prime},\,\,\,\varpi_{7} = \varphi ,\,\,\,\varpi_{8} = \varphi^{\prime}. $$

By employing Eq. (21) in Eq. (17), and Eqs. (13–16), we get:

22 $$ \begin{gathered} \left( {1 + \frac{1}{\beta }} \right)\left[ {\varpi^{\prime}_{4} + \frac{{2\varpi_{4} }}{\eta + \kappa } - \frac{{\varpi_{3} }}{{\left( {\eta + \kappa } \right)^{2} }} + \frac{{\varpi_{2} }}{{\left( {\eta + \kappa } \right)^{3} }}} \right] + We\left[ \begin{gathered} \left( {\varpi_{3} \varpi^{\prime}_{4} + \hbar_{3}^{2} } \right) - \frac{1}{{\left( {\eta + \kappa } \right)^{2} }}\left( {\varpi_{2} \varpi^{\prime}_{4} - 2\varpi_{3} \varpi_{4} } \right) - \frac{2}{{\left( {\eta + \kappa } \right)^{2} }} \hfill \\ \left( {\varpi_{3}^{2} + \varpi_{2} \varpi_{4} } \right) + \frac{{4\varpi_{2} \varpi_{3} }}{{\left( {\eta + \kappa } \right)^{3} }} - \frac{{2\varpi_{2}^{2} }}{{\left( {\eta + \kappa } \right)^{4} }} \hfill \\ \end{gathered} \right] \hfill \\ + \frac{\kappa }{\eta + \kappa }\left[ \begin{gathered} \varpi_{1} \varpi_{4} - \hfill \\ \varpi_{2} v_{3} \hfill \\ \end{gathered} \right] + \frac{\kappa }{{\left( {\eta + \kappa } \right)^{2} }}\left[ \begin{gathered} \varpi_{1} \varpi_{3} \hfill \\ - \varpi_{2}^{2} \hfill \\ \end{gathered} \right] - \frac{\kappa }{{\left( {\eta + \kappa } \right)^{3} }}\varpi_{1} \varpi_{2} - \delta^{*} \left( { \left( {\frac{\eta }{2}} \right)\varpi_{4} + \frac{{3\varpi_{3} }}{2}} \right) - \frac{{\delta^{*} }}{{\left( {\eta + \kappa } \right)}}\left[ {\left( {\frac{\eta }{2}} \right)\varpi_{3} + \varpi_{2} } \right] \hfill \\ - \frac{{2\beta_{m} }}{{\left( {\eta + b} \right)^{4} }}\left[ { \left( {\frac{1}{\eta + \kappa } - \frac{4}{\eta + b}} \right)\varpi_{5} + \varpi_{6} } \right] + \lambda_{T} \left[ {\frac{{\varpi_{5} }}{\eta + \kappa } + \varpi_{6} } \right] - M^{*} \left[ {\varpi_{3} + \frac{{\varpi_{2} }}{\eta + \kappa }} \right] = 0. \hfill \\ \end{gathered} $$

23 $$ \left. \begin{gathered} \frac{1}{Pr}\left[ {1 + Rd} \right]\left( { \varpi^{\prime}_{6} + \left( {\frac{1}{\eta + \kappa }} \right)\varpi_{6} } \right) + \frac{\kappa }{\eta + \kappa }\varpi_{1} \varpi_{6} + Nb \varpi_{8} \varpi_{6} + Nt \left( {\varpi_{6} } \right)^{2} - \left( {\frac{\eta }{2}} \right)\delta^{*} \varpi_{6} + Q \varpi_{5} e^{ - \eta } + Ec M^{*} \left( {\varpi_{2} } \right)^{2} + D_{f} \hfill \\ \left( {\varpi^{\prime}_{8} + \frac{1}{\eta + K}\varpi_{8} } \right) - C_{H} \left( {\frac{\kappa }{\eta + \kappa }} \right)^{2} \left[ \begin{gathered} \varpi_{1}^{2} \varpi^{\prime}_{6} + \varpi_{1} \varpi_{2} \varpi_{6} \hfill \\ - \left( {\frac{{\varpi_{1}^{2} }}{\eta + \kappa }} \right)\theta^{\prime} \hfill \\ \end{gathered} \right] + \frac{2}{Pr}\frac{{\beta_{m} \lambda_{m} \left( {\varpi_{5} - \varepsilon } \right)}}{{\left( {\eta + b} \right)^{3} }}\left[ {\frac{{K\varpi_{1} }}{{\left( {\eta + \kappa } \right)}}\left\{ {1 - \frac{2}{{\left( {\eta + b} \right)^{2} }}} \right\} - \frac{{\varpi_{2} }}{\eta + b}} \right] = 0, \hfill \\ \end{gathered} \right\} $$

24 $$ \varpi^{\prime}_{8} + \frac{1}{\eta + K}\varpi_{8} + Sc\left( {\frac{K}{\eta + K}\varpi_{1} \varpi_{8} - K_{r} \left( {1 + \omega \varpi_{5} } \right)^{n} Exp\left( { - \frac{{E_{1} }}{{1 + \omega \varpi_{5} }}} \right) + \left( {\varpi^{\prime}_{6} + \frac{1}{\eta + K}\varpi_{6} } \right)Sr} \right) = 0. $$

The transfused I & BCs are:

25 $$ \left. \begin{gathered} \varpi_{2} = 1 + L_{1} \left( { \hbar_{3} - \left( {\frac{1}{\kappa }} \right)\hbar_{2} } \right), M_{e} \,\varpi_{6} + Pr\,\varpi_{1} = 0, \varpi_{5} = 1, \varpi_{7} \left( 0 \right) = 1, \, \,{\mathrm{at}} \,\,\eta = 0 \hfill \\ \varpi_{2} \to 0, \,\,\,\varpi_{3} \to 0, \,\,\,\varpi_{5} \to 0,\,\,\, \varpi_{7} \left( \infty \right) \to 1 \,\,\,{\mathrm{as}}\,\,\, \eta \to \infty . \hfill \\ \end{gathered} \right\} $$

### ANN interpretation

To augment the analytical framework of the current model, an Artificial Neural Network (ANN) using the robust optimization capabilities of the Levenberg–Marquardt Algorithm (LMA) has been implemented. The successful deployment of this intelligent computing paradigm is predicated on the achievement of a fundamental dataset, which, in this study, is meticulously generated via the bvp4c numerical technique. This numerical method furnishes the comprehensive reference solutions that serve as the target data for the neural network's supervised learning phase. Subsequently, this foundational dataset is utilized to train the ANN through an iterative process involving multiple epochs and repeated exposures to the data, thereby enabling the network to learn and approximate the complex underlying dynamics inherent in the system. This rigorous training regimen, driven by the efficiency of the LMA in minimizing the mean squared error between the network predictions and the bvp4c-derived targets, ultimately yields a robust and accurate ANN model capable of effectively generalizing the solution across the parameter space.

## Discussion of results

The choice of the combined WC rheological model is motivated physically as it is the only model that can describe material whose properties exhibit both yield stress and pseudoplastic (shear-thinning) behavior. The Casson model defines a particular yield stress value below which the suspension is not flowing, which is a good approximation to the structured network columns observed in complex suspensions. After exceeding this initial yield stress, mechanical forces cause the effective viscosity to decrease in a manner exactly predicted by Williamson model at high shear stretching rates. This is a combination formulation, which is very suitable for the simulation of complex real-world engineering and clinical systems. For advanced industrial processing, it is an effective tool for modelling the optimization of polymer extrusion dies, paint formulations and synthetic lubricant coatings, where fluids need to be capable of running under gravity but flow smoothly in continuous mechanical stretching. The same is true in biomedical engineering: human blood is shear-thinning under high velocity in the micro scale arteries and has prominent Williamson shear-thinning characteristics while it takes the form of a Casson suspension under low shear conditions.

This study has systematically examined the Williamson-Casson nanofluid flow over a slippery curved expanding surface while simultaneously incorporating the Cattaneo-Christov heat flux model and melting heat transfer conditions, particularly through the application of machine learning algorithms. To address this deficiency, the present study undertakes a comprehensive investigation of the coupled nonlinear transport phenomena governing such fluid behavior by using various effects. The flow is affected by melting heat transfer conditions at boundary, activation energy, chemical reactivity and Soret/Dufour effects. The modeled equations have solved through bvp4c approach in dimensionless form. The solution obtained from this approach is then used to provide a dataset for ANN approach. The effects of various parameters on different profiles are examined in following lines.

### Velocity profiles

**Fig. 2:**
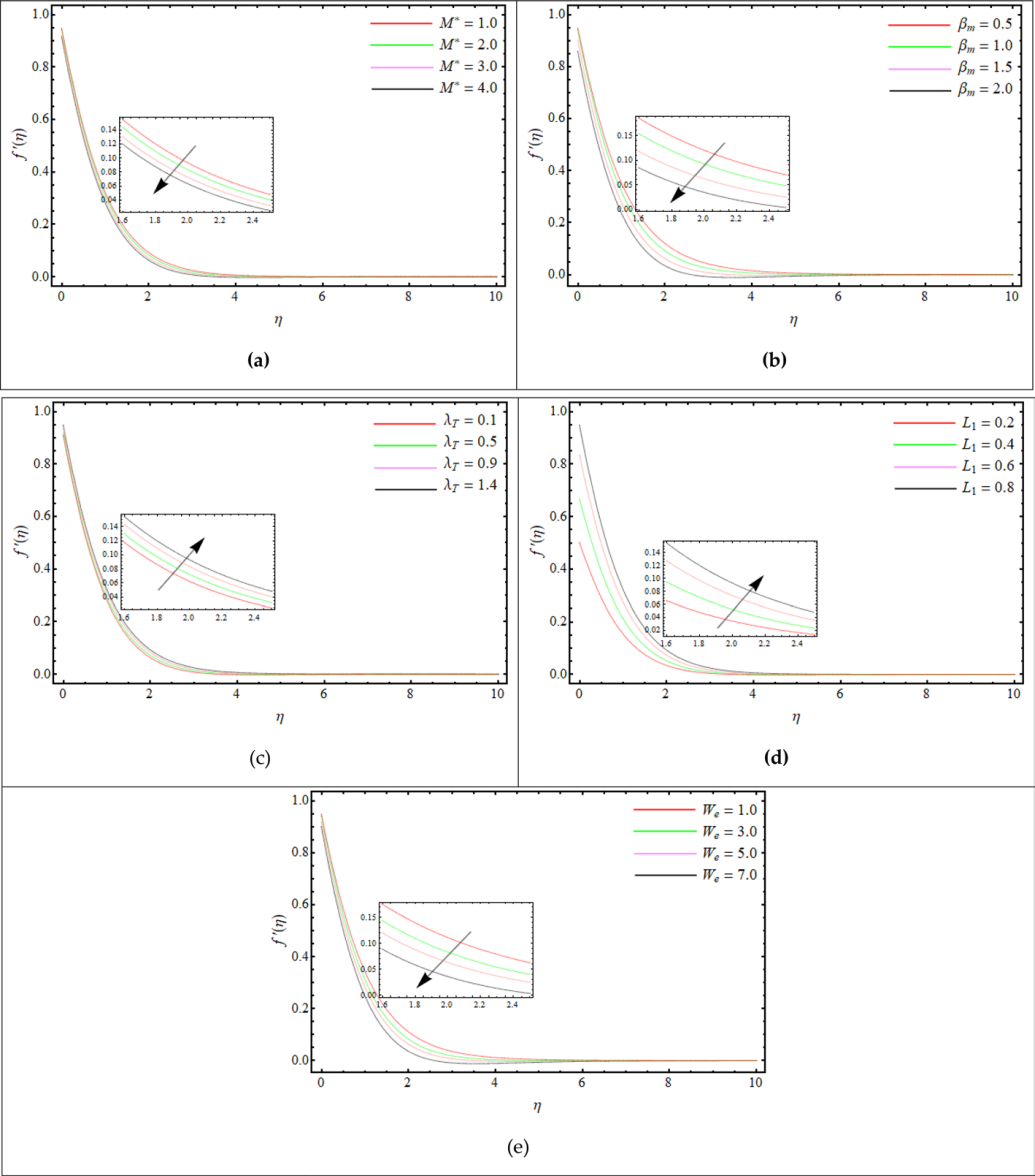
Effect of **a** magnetic parameter $M^{*}$, **b** ferrohydrodynamic interaction $\beta_{m}$, **c** thermal buoyancy factor $\lambda_{T}$, **d** velocity slip $L_{1}$, and **e** Weissenberg number $We$, on the velocity profile $f^{\prime}\left( \eta \right)$ respectively

The effect of various parameters on velocity profiles $\left\{ {f^{\prime}\left( \eta \right)} \right\}$ is explained in Fig. 2a–e. Figure 2a portrays that with growth in magnetic parameter $\left( {M^{*} } \right)$ there is decline in $\left\{ {f^{\prime}\left( \eta \right)} \right\}$. Physically, with growth in $\left( {M^{*} } \right)$ the retarding effect of $\left\{ {f^{\prime}\left( \eta \right)} \right\}$ is fundamentally a consequence of the Lorentz force. As $\left( {M^{*} } \right)$ rises, the conductive fluid experiences a stronger electromagnetic interaction on surface of curved sheet. The motion of the fluid across the magnetic field lines induces an electromotive force, which in turn generates induced currents. These currents interact with the applied magnetic field to produce a Lorentz force that acts in the direction opposite to the fluid's motion. This body force effectively increases the fluid's resistance to flow, manifesting as a flattening of the velocity profile and a significant reduction in the bulk fluid velocity, particularly in the core region away from the boundaries. Figure 2b portrays that with growth in ferro-hydrodynamic interaction $\left( {\beta_{m} } \right)$, there is decline in $\left\{ {f^{\prime}\left( \eta \right)} \right\}$. With the intensification of ferrohydrodynamic interaction $\left( {\beta_{m} } \right)$, the fluid experiences a stronger coupling between the applied magnetic field and the magnetizable nanoparticles suspended within it. Physically, this interaction gives rise to enhanced magnetic body forces (Kelvin forces) and Lorentz-type resistive effects that act opposite to the primary flow direction. As magnetic field strength increases, the alignment and magnetization of particles augment the internal resistance of the fluid, effectively increasing its apparent viscosity and damping momentum transport. Consequently, the kinetic energy of the fluid is partially converted into magnetic energy and dissipated through internal friction, leading to a suppression of boundary-layer thickness and a marked reduction in velocity profiles $\left\{ {f^{\prime}\left( \eta \right)} \right\}$. This deceleration reflects the dominant retarding influence of magnetic forces over inertial forces within the flow regime. Figure 2c portrays that with growth in thermal buoyancy factor $\left( {\lambda_{T} } \right)$ there is augmentation in $\left\{ {f^{\prime}\left( \eta \right)} \right\}$. An intensification in $\left( {\lambda_{T} } \right)$ physically signifies the strengthening of natural convection forces generated due to temperature-induced density variations within the fluid. When the buoyancy parameter $\left( {\lambda_{T} } \right)$ grows, the temperature difference between the fluid and the ambient medium becomes more influential, producing a larger upward body force in the momentum equation. This enhanced buoyant force assists with the primary flow direction, reduces the effective resistance offered by viscous forces, and accelerates fluid particles within the boundary layer. Consequently, the velocity profiles $\left\{ {f^{\prime}\left( \eta \right)} \right\}$ exhibits augmentation because the thermal energy is being more efficiently converted into kinetic energy, leading to a thicker momentum boundary layer and intensified fluid motion. Figure 2d portrays that with growth in velocity slip parameter $\left( {L_{1} } \right)$ there is augmentation in $\left\{ {f^{\prime}\left( \eta \right)} \right\}$. Actually for fluid flow on curved surface, the augmentation of velocity profiles $\left\{ {f^{\prime}\left( \eta \right)} \right\}$ with an increasing velocity slip parameter $\left( {L_{1} } \right)$ is a direct consequence of the reduction in frictional resistance at the solid–fluid boundary. The slip parameter quantifies the extent to which the fluid molecules can slide along the wall rather than adhering strictly to the no-slip condition. As this parameter grows, the wall no longer enforces a zero-velocity constraint; consequently, the retarding shear stress transmitted from the stationary boundary to the fluid layers above is diminished. This reduction in boundary drag allows the bulk fluid to move more freely, resulting in a higher velocity $\left\{ {f^{\prime}\left( \eta \right)} \right\}$ at the wall itself and a substantial overall increase in the flow rate, as the velocity profile shifts upward across the curved surface. Figure 2e portrays that with growth in Weissenberg number $\left( {We} \right)$ there is reduction in $\left\{ {f^{\prime}\left( \eta \right)} \right\}$. The reduction in $\left\{ {f^{\prime}\left( \eta \right)} \right\}$ with an increasing $\left( {We} \right)$ in flow over a curved stretching surface is a manifestation of the fluid's growing elastic resistance to extensional deformation imposed by the curved geometry. The Weissenberg number $\left( {We} \right)$ compares the elastic relaxation time of the polymer chains to the characteristic flow time, and as it rises, the fluid behaves less like a simple viscous liquid and more like a viscoelastic solid. On a curved surface, the stretching generates not only a tangential boundary layer but also a substantial normal stress difference due to the curvature-induced centrifugal effects and streamline tension. At higher $\left( {We} \right)$, the strained polymer chains generate large opposing normal stresses (hoop stresses) that act against the radial stretching motion. This elastic stiffness effectively retards the flow, requiring more energy to deform the fluid, which manifests as a suppression of the velocity field as examined in Fig. 2e.

### Thermal profiles

**Fig. 3:**
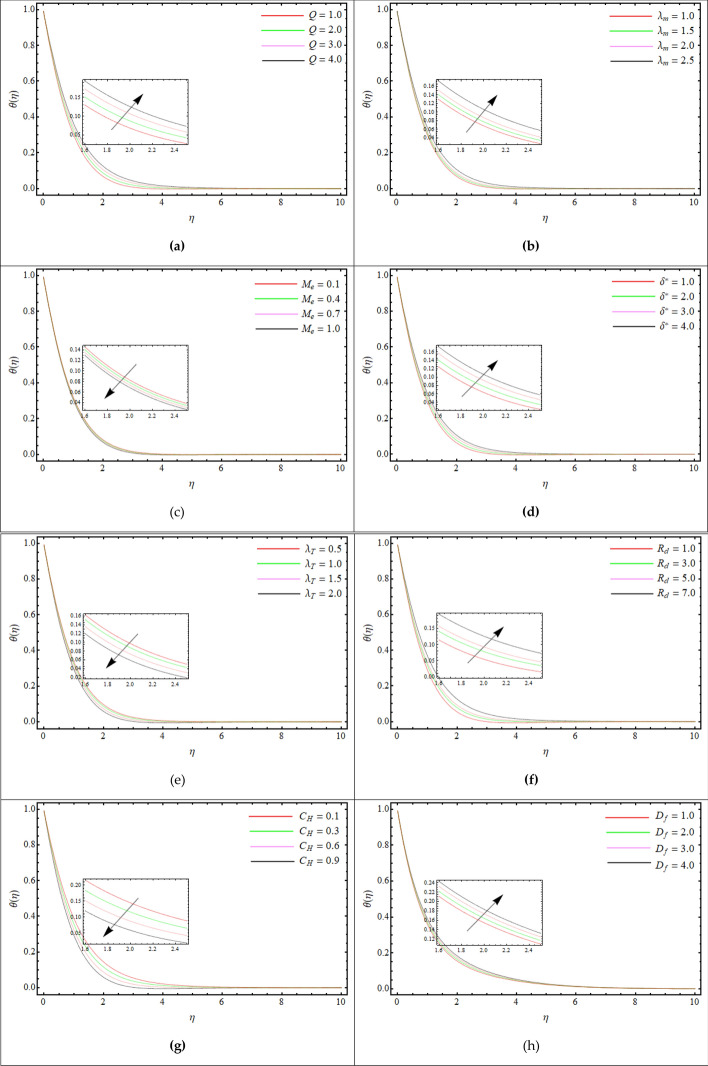
Effect of **a** heat source/sink parameter *Q*, **b** heat dissipation factor $\lambda_{m}$, **c** melting heat parameter $M_{e}$, **d** unsteadiness factor $\delta^{*}$, **e** thermal buoyancy factor $\lambda_{T}$, **f** heat radiation factor $R_{d}$, **g** thermal relaxation factor $C_{H}$, and **h** Dufour number $D_{f}$ on the temperature field $\theta \left( \eta \right)$ respectively

The effect of various parameters on thermal profiles $\left\{ {\theta \left( \eta \right)} \right\}$ is explained in Fig. 3a–h. Figure 3a portrays that with growth in heat source/sink parameter $\left( Q \right)$ there is augmentation in $\left\{ {\theta \left( \eta \right)} \right\}$. The augmentation of $\left\{ {\theta \left( \eta \right)} \right\}$ with an increasing $\left( Q \right)$ on a curved stretching surface is physically interpreted as the manifestation of additional internal heat energy generation within the fluid. A positive heat source parameter acts as a volumetric energy input, directly increasing the local temperature of the fluid particles as they traverse the curved surface. This internal heating supplements the thermal energy conducted from the heated stretching surface, leading to a thicker thermal layer at boundary and higher overall fluid temperatures. Further, the curved nature of the surface influences this effect by altering the velocity and pressure distribution, but the primary physical mechanism is the direct addition of heat energy within the fluid bulk, which naturally results in elevated temperature fields. Figure 3b describes that with growth in heat dissipation factor $\left( {\lambda_{m} } \right)$ there is augmentation in $\left\{ {\theta \left( \eta \right)} \right\}$. The enhancement of $\left\{ {\theta \left( \eta \right)} \right\}$ with an increasing heat dissipation factor $\left( {\lambda_{m} } \right)$ is physically interpreted as the conversion of mechanical energy into thermal energy due to viscous friction inside the fluid. As the curved stretching surface deforms, it generates significant velocity gradients, particularly in the region near the wall. The heat dissipation factor, representing the viscous dissipation effect, quantifies the irreversible work done by these viscous stresses, which heats the fluid internally. This process is amplified on a curved surface because the centrifugal forces and curvature-induced pressure variations create additional resistance to flow, requiring more work to overcome the fluid’s viscosity. The mechanical energy lost in this manner is transformed into internal heat energy, acting as a distributed heat source that elevates the fluid temperature, thereby augmenting the thermal boundary layer width and the hence temperature distribution of the flow on curved surface. Figure 3c describes that with growth in melting heat parameter $\left( {M_{e} } \right)$, there is reduction in $\left\{ {\theta \left( \eta \right)} \right\}$. The reduction in $\left\{ {\theta \left( \eta \right)} \right\}$ with an increasing $\left( {M_{e} } \right)$ is physically interpreted as the absorption of latent heat required to facilitate the phase change from solid to liquid at the boundary. In this process, a portion of the thermal energy that would otherwise elevate the temperature of the fluid is diverted to overcome the enthalpy of fusion, effectively consuming heat to convert the solid medium into a liquid state. As the melting parameter $\left( {M_{e} } \right)$ grows, a greater amount of heat is absorbed from the curved stretching surface and the adjacent fluid layers to sustain this phase transition. This absorption acts as a thermal sink near the boundary, lowering the peak temperatures and reducing the thermal layer width at borderline. Consequently, the temperature distribution $\left\{ {\theta \left( \eta \right)} \right\}$ of fluid decreases as more energy is allocated to the melting process rather than to sensible heat increase. Figure 3d describes that with growth in unsteadiness factor $\left( {\delta^{*} } \right)$, there is augmentation in $\left\{ {\theta \left( \eta \right)} \right\}$. The augmentation of $\left\{ {\theta \left( \eta \right)} \right\}$ with an increasing $\left( {\delta^{*} } \right)$ is physically interpreted as the reduced time available for thermal diffusion relative to the accelerating fluid motion. As $\left( {\delta^{*} } \right)$ grows, the stretching rate of the curved surface intensifies over time, causing the fluid particles to be driven away from the boundary more rapidly. This accelerated outward motion inhibits the efficient diffusion of heat away from the hot surface into the cooler ambient fluid, causing thermal energy to accumulate in the near-wall region. Furthermore, the sudden temporal changes in the flow field generate additional shear stresses and velocity gradients, which enhance frictional heating through viscous dissipation. On a curved geometry, this effect is compounded by the centrifugal forces that push fluid outward, further restricting the vertical propagation of thermal energy while simultaneously trapping heat near the curved wall, resulting in elevated temperature distributions $\left\{ {\theta \left( \eta \right)} \right\}$ throughout the flow domain on curved surface. Figure 3e describes that with growth in thermal buoyancy factor $\left( {\lambda_{T} } \right)$, there is decline in $\left\{ {\theta \left( \eta \right)} \right\}$. The decline in $\left\{ {\theta \left( \eta \right)} \right\}$ with an increasing $\left( {\lambda_{T} } \right)$ is physically interpreted as the enhancement of convective cooling driven by buoyancy-induced fluid acceleration. As $\left( {\lambda_{T} } \right)$ grows, the temperature difference between the heated curved surface and the ambient fluid generates stronger buoyancy forces, which act to accelerate the fluid motion in the vertical direction against the retarding effects of viscosity. This accelerated flow enhances the rate of heat transfer away from the surface by increasing the convective transport of thermal energy into the cooler ambient region. On the curved surface, the centrifugal forces interact with these buoyancy currents to create more vigorous mixing and fluid circulation near the wall, which efficiently sweeps away the heated fluid and replaces it with cooler fluid from the surroundings. Consequently, the thermal boundary layer becomes thinner, and the temperature distribution $\left\{ {\theta \left( \eta \right)} \right\}$ of flow decreases as more heat is effectively carried away by the strengthened buoyancy-driven convection currents. Figure 3f describes that with growth in radiation factor $\left( {R_{d} } \right)$, there is a surge in $\left\{ {\theta \left( \eta \right)} \right\}$. The augmentation of $\left\{ {\theta \left( \eta \right)} \right\}$ with an increasing $\left( {R_{d} } \right)$ is physically interpreted as the additional heat transfer mechanism provided by thermal radiation, which supplements the conductive and convective modes of energy transport. As $\left( {R_{d} } \right)$ grows, the fluid becomes more optically thick and become capable of emitting and absorbing radiative energy, meaning that heat is not only transferred through direct fluid contact but also through electromagnetic wave propagation. This radiative transfer allows thermal energy to penetrate deeper into the fluid layers and be absorbed by the medium, effectively acting as a distributed energy source throughout the thermal boundary layer. On the curved stretching surface, the curvature influences the view factors and the directional distribution of radiative heat flux, but the fundamental effect remains that radiation adds an extra pathway for heat to enter and accumulate within the fluid. Since radiative transfer typically becomes more substantial at higher temperatures, the presence of strong radiation effects traps more energy within the flow domain, leading to elevated temperature as explained in Fig. 3f. Figure 3g describes that with growth in thermal relaxation factor $\left( {C_{H} } \right)$, there is decline in $\left\{ {\theta \left( \eta \right)} \right\}$. The decline in $\left\{ {\theta \left( \eta \right)} \right\}$ with an increasing $\left( {C_{H} } \right)$ is physically interpreted as the manifestation of a delayed thermal response within the fluid medium. Unlike classical heat conduction, where temperature changes propagate instantaneously, a fluid with a substantial thermal relaxation parameter requires a finite time for heat flux to establish itself in response to a temperature gradient. This means that as the fluid particles move rapidly along the curved stretching surface, they are carried away from the heated boundary before they have had sufficient time to fully absorb and conduct the thermal energy from the wall. On the curved surface, where centrifugal forces accelerate the fluid away from the surface, this retardation effect is amplified, and the fluid escapes the heating zone faster than the thermal wave that can propagate into it. Consequently, less heat penetrates the fluid layers, resulting in a thinner thermal layer at boundary and reduced temperature distribution $\left\{ {\theta \left( \eta \right)} \right\}$. Figure 3h describes that with growth in Dufour number $\left( {D_{f} } \right)$ there is augmentation in $\left\{ {\theta \left( \eta \right)} \right\}$. The augmentation of $\left\{ {\theta \left( \eta \right)} \right\}$ with an increasing $\left( {D_{f} } \right)$ is physically interpreted as the energy flux generated by concentration gradients of fluid, representing a cross-diffusion phenomenon. As species diffuse from regions of high concentration to low concentration due to the stretching surface boundary conditions, this mass transfer process carries with it an associated flux of thermal energy. The Dufour effect captures this coupling, where the chemical potential differences drive not only the redistribution of species but also the transport of sensible heat into regions of lower concentration. As $\left( {D_{f} } \right)$ grows, this energy transport mechanism becomes more efficient, with a greater proportion of the concentration gradient work being converted into thermal energy within the fluid. This effectively acts as an additional distributed heat source that elevates the local temperature beyond what would be expected from thermal diffusion alone, leading to enhanced thermal boundary layer thickness and elevated temperature distributions throughout the flow domain on surface.

### Concentration profiles

**Fig. 4:**
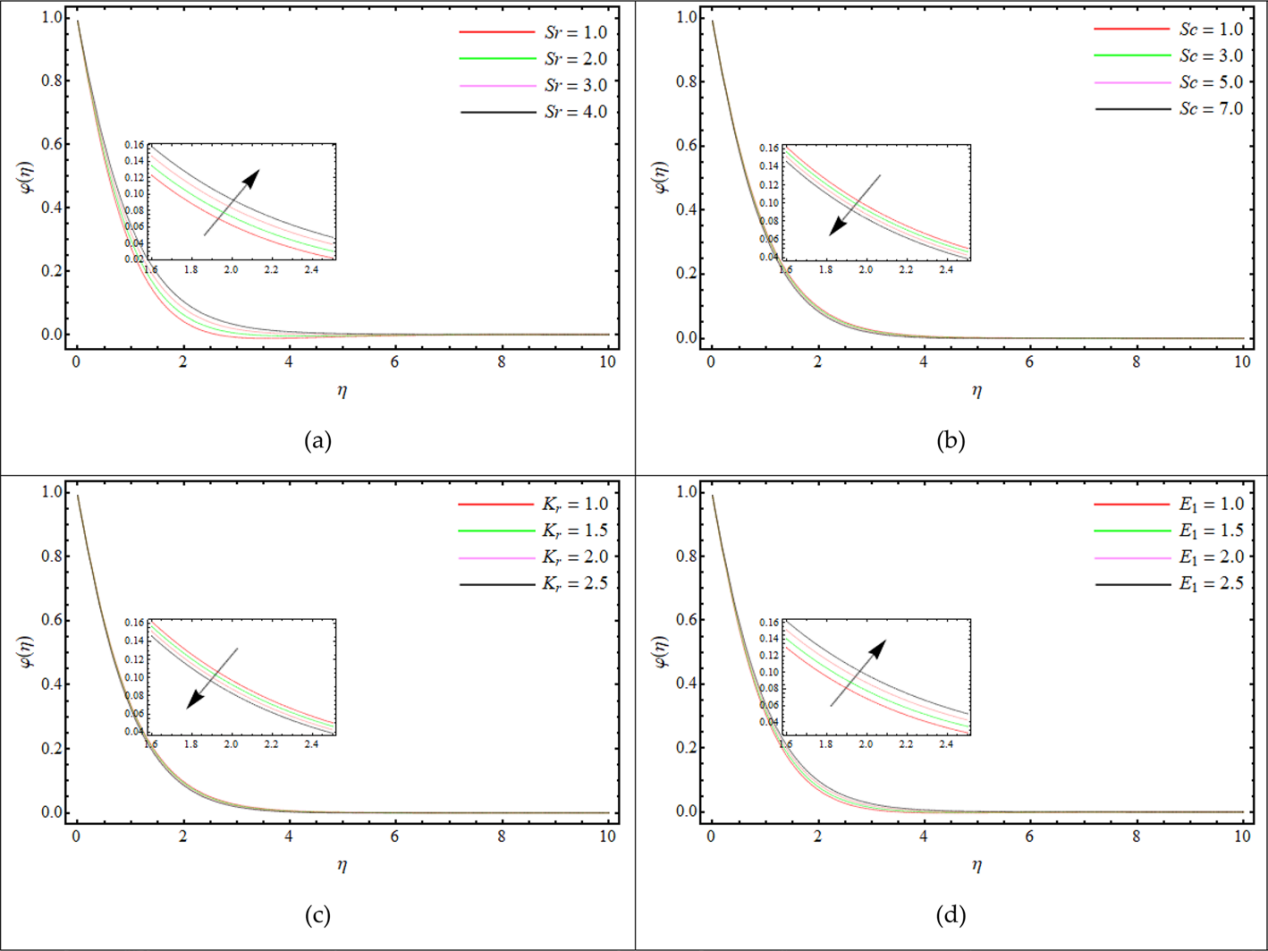
Effect of **a** Soret number *Sr*, **b** Schmidt number $Sc$, **c** chemical reaction rate $K_{r}$, and **d** activation energy $E_{1}$, on the fluid concentration $\varphi \left( \eta \right)$ respectively

The effect of various parameters on concentration profiles $\left\{ {\varphi \left( \eta \right)} \right\}$ is explained in Fig. 4a–d. Figure 4a portrays that with growth in Soret number $\left( {Sr} \right)$ there is augmentation in $\left\{ {\varphi \left( \eta \right)} \right\}$. The augmentation of $\left\{ {\varphi \left( \eta \right)} \right\}$ with an increasing $\left( {Sr} \right)$ is physically interpreted as the species separation driven by temperature gradients within the fluid, representing the inverse cross-diffusion phenomenon to the Dufour effect. As thermal energy propagates from the heated curved stretching surface into the cooler fluid layers, the resulting temperature gradient creates a thermodynamic force that induces lighter species to migrate toward hotter regions and heavier species toward cooler regions through thermal diffusion. The Soret parameter quantifies the strength of this thermophoretic separation, where each unit of temperature difference generates a proportional flux of chemical species independent of concentration gradients. On the curved stretching surface, the curvature-enhanced thermal boundary layer provides steeper temperature gradients near the wall, which, when coupled with a growing Soret number, drives more vigorous thermally induced mass transport. This thermo-diffusion mechanism effectively pumps species into regions where they would not otherwise accumulate based solely on Fickian diffusion, acting as a distributed source that elevates the local species’ concentration. Consequently, the concentration layer thickens at boundary and the overall species distribution increases as thermal energy gradients are increasingly converted into chemical potential gradients through this cross-coupling effect. Figure 4b portrays that with growth in Schmidt number $\left( {Sc} \right)$, there is reduction in $\left\{ {\varphi \left( \eta \right)} \right\}$. The reduction in $\left\{ {\varphi \left( \eta \right)} \right\}$ with an increasing $\left( {Sc} \right)$ is physically interpreted as the diminished molecular diffusivity of species relative to the momentum diffusivity of the fluid. As $\left( {Sc} \right)$ grows, the species particles struggle to diffuse away from the curved surface compared to how quickly the fluid motion carries them downstream. On the curved surface, the stretching-induced velocity field and centrifugal forces create strong convective transport parallel to the surface, but the high Schmidt number limits the ability of species to migrate perpendicularly into the fluid layers. This results in a thinner concentration boundary layer where dissolved species remain confined near the wall rather than penetrating deep into the flow domain. The physical consequence is that mass transfer becomes increasingly dominated by the diffusive mechanism relative to the vigorous convective motion, causing the concentration distribution $\left\{ {\varphi \left( \eta \right)} \right\}$ to decay more rapidly with distance from the surface and reducing the overall species penetration depth. Figure 4c portrays that with growth in chemical reaction rate $\left( {K_{r} } \right)$, there is reduction in $\left\{ {\varphi \left( \eta \right)} \right\}$. The reduction in $\left\{ {\varphi \left( \eta \right)} \right\}$ with an increasing $\left( {K_{r} } \right)$ is physically interpreted as the consumption of chemical species through reactive transformation as they travel along the curved stretching surface. As $\left( {K_{r} } \right)$ grows, the characteristic time scale for chemical conversion becomes much shorter than the time scale for mass diffusion, meaning that species molecules are rapidly transformed into products before they have the opportunity to diffuse deeper into the fluid layers. This consumption mechanism acts as a distributed sink term throughout the concentration boundary layer, removing species mass at a rate proportional to the local concentration itself. Consequently, the penetration depth of the undisturbed species is severely limited, the concentration boundary layer thins substantially, and the overall species distribution decays more rapidly with distance from the surface compared to cases with slower chemical reaction, where species can survive longer and diffuse farther into the flow domain. Figure 4d portrays that with growth in activation energy $\left( {E_{1} } \right)$ there is augmentation in $\left\{ {\varphi \left( \eta \right)} \right\}$. The augmentation of $\left\{ {\varphi \left( \eta \right)} \right\}$ with an increasing $\left( {E_{1} } \right)$ is physically interpreted as the reduced rate of species consumption due to the heightened energy barrier required for chemical reaction to occur. In temperature-dependent reaction kinetics, the Arrhenius relationship dictates that a higher activation energy makes the reaction rate exponentially more sensitive to temperature. At a given thermal condition, fewer molecules possess the necessary kinetic energy to overcome this barrier and undergo transformation. As this parameter grows, the chemical reaction proceeds much more slowly even when species are present in regions with favorable temperatures, effectively weakening the destructive sink term that would consume the diffusing species. This slower kinetics allows species to penetrate deeper into the thermal layer at boundary, accumulate in regions where they would have been consumed under lower activation energy conditions, and ultimately maintain higher concentration levels throughout the flow domain compared as explains in Fig. 4d

### ANN graphs analysis

**Fig. 5:**
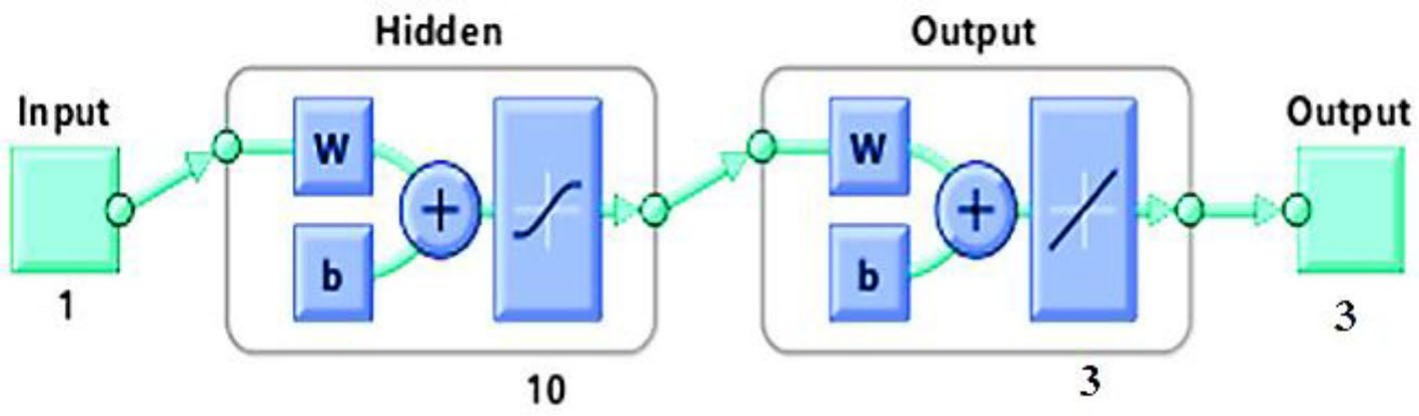
Network architecture diagram for LMA model

In this study, the LMA has applied to a dataset generated via bvp4c approach. A neural network architecture with a single hidden layer containing 10 neurons was constructed for this purpose. The dataset was randomly partitioned into three subsets for model development: 70% for training, 15% for testing, and 15% for validation. The overall structure of the LMA model, including its input, hidden, and output layers and the use of Mean Squared Error (MSE) for performance evaluation, is schematically represented in Fig. 5.

The detailed hyperparameter tuning test was performed to make sure the Artificial Neural Network (ANN) was not underspecified for our coupled fluid system, and thus the number of hidden neurons was varied from 2 to 30. The outcome of this sensitivity analysis showed that the sharp velocity and thermal boundary layer drops were difficult to map for fewer than 10 neurons, but more than 10 had no noticeable effect on the accuracy of prediction. Rather, the larger number of neurons tended to take much longer to train and led to higher likelihood of overfitting the basic data set. The best results were achieved by using just 10 hidden neurons. It offers a clean, well-defined architecture that is simple to operate, while maintaining a very low profile of mean squared error (10⁻⁷), and excellent regression values (R^2^ ≈ 1.0000).

**Fig. 6:**
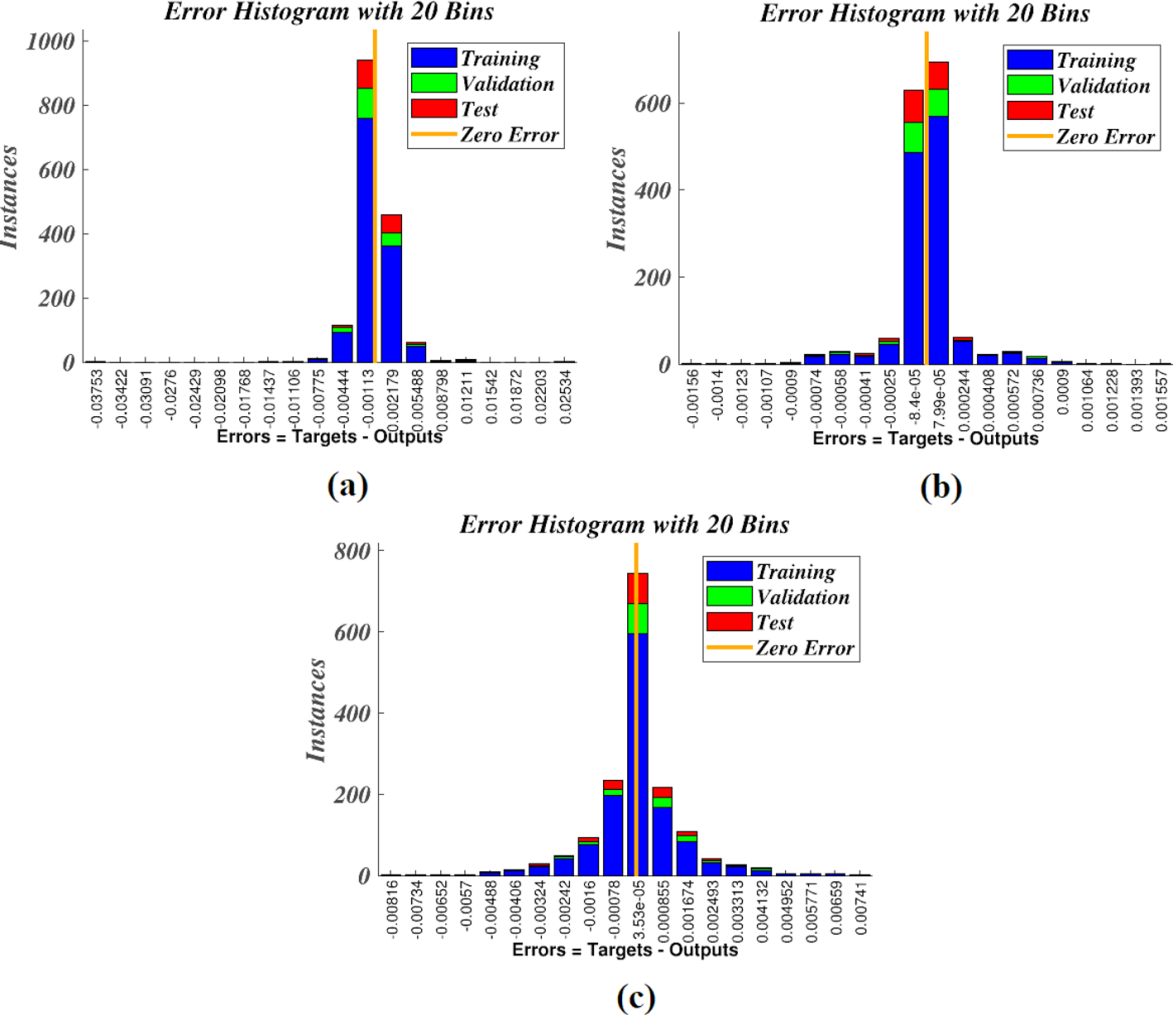
Error histogram

**Fig. 7:**
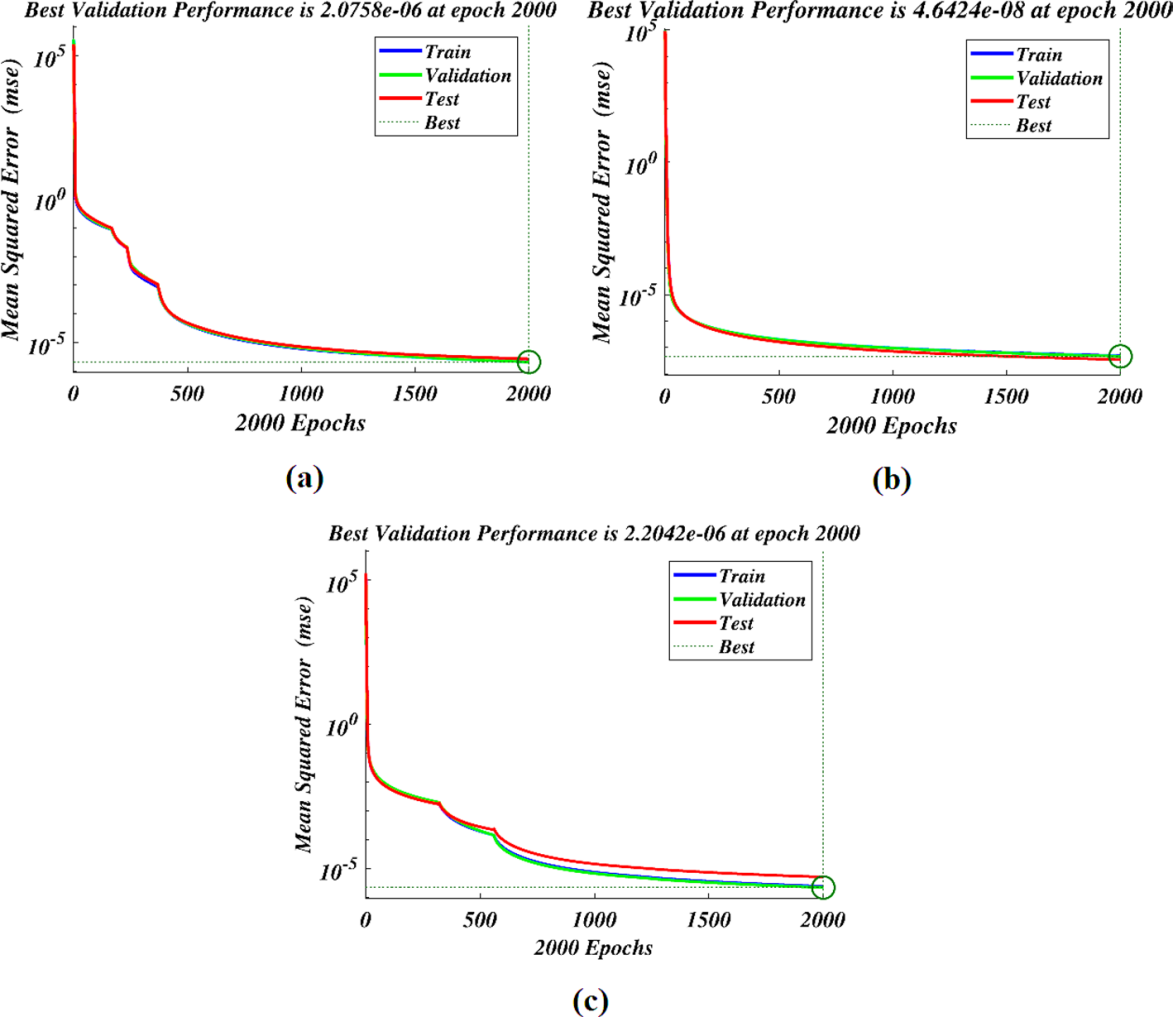
Mean squared error

**Fig. 8:**
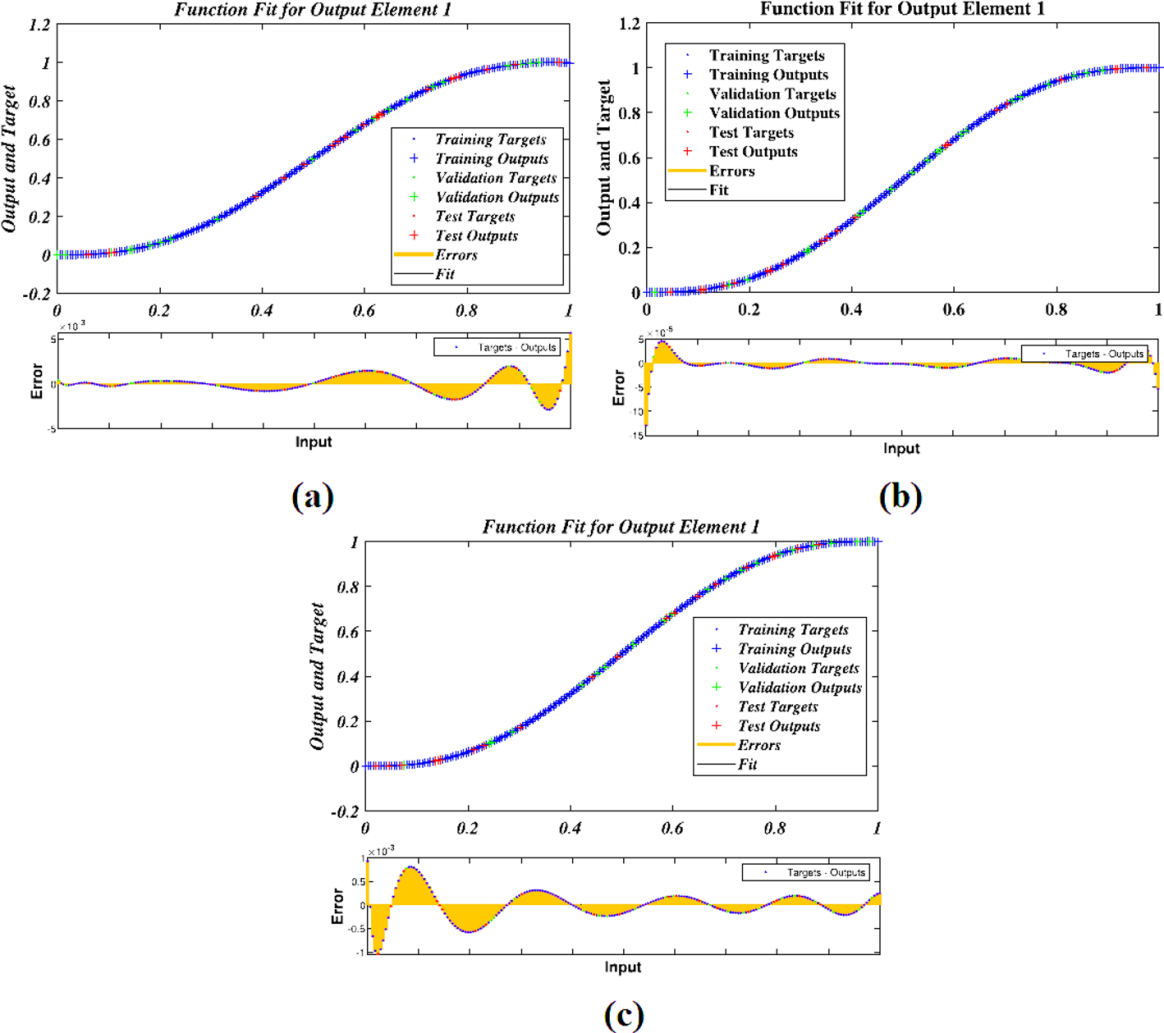
Function fit graph

**Fig. 9:**
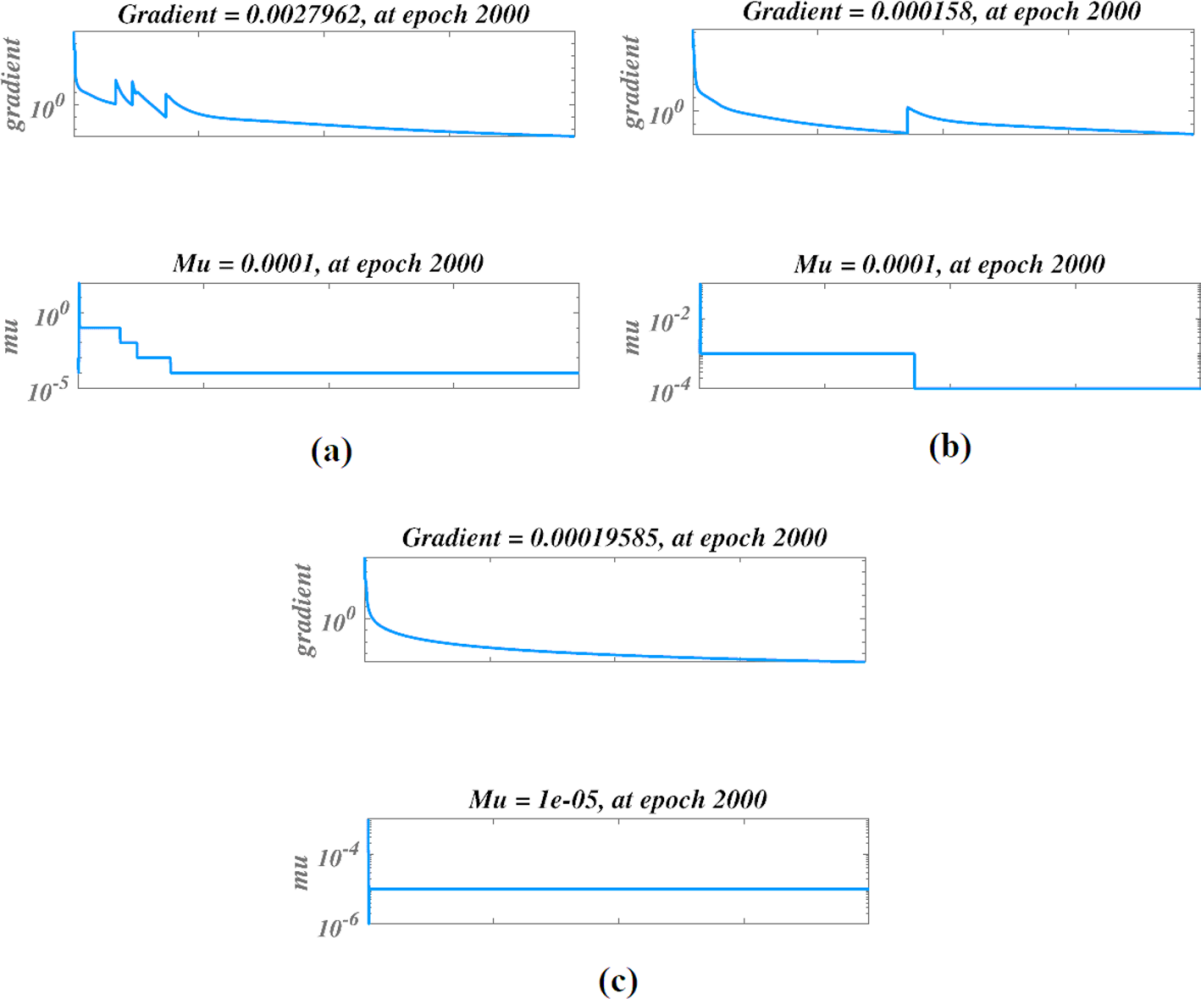
Training states

**Fig. 10:**
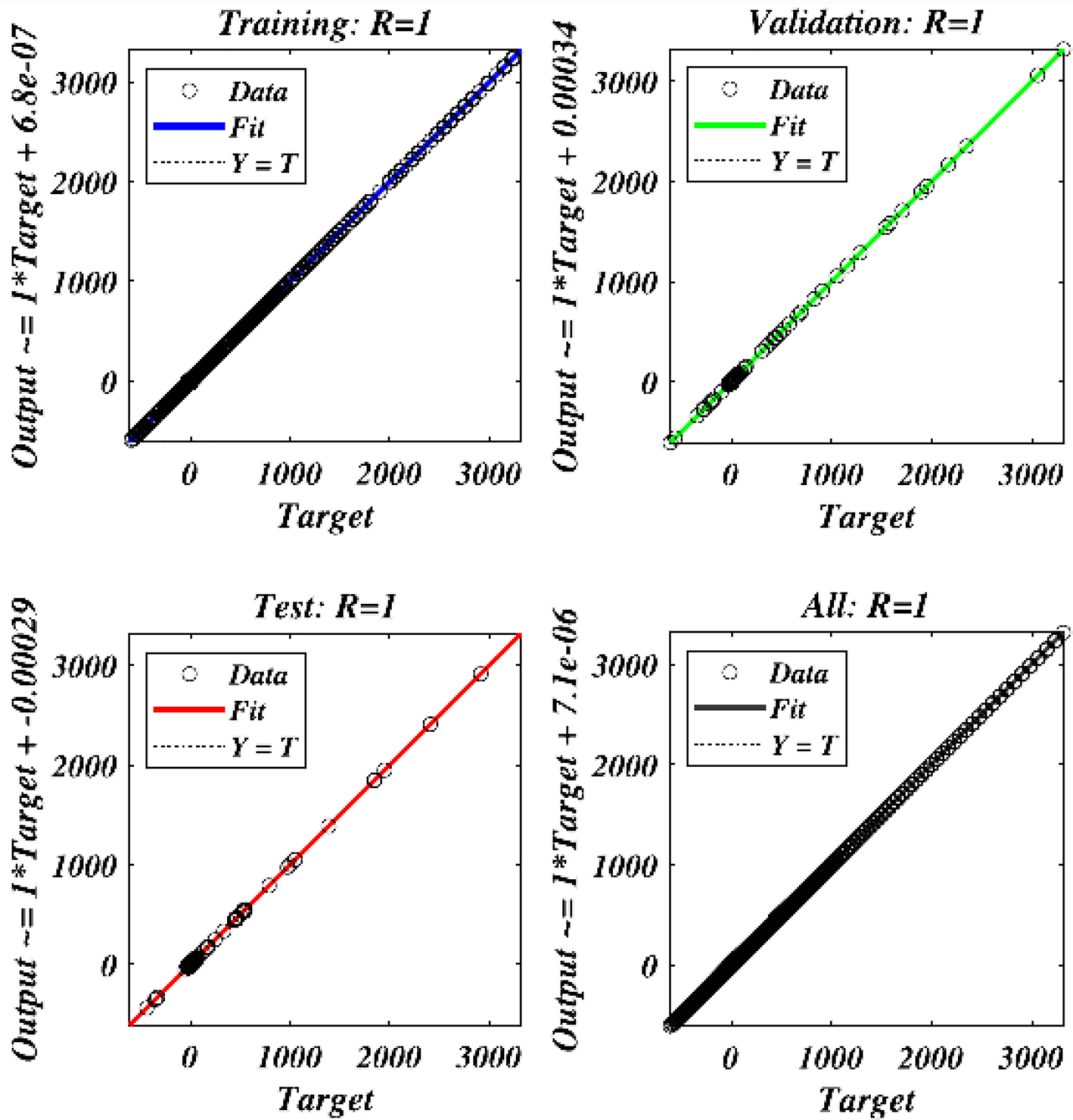
Regression analysis (Case-1)

A visual assessment of the model's predictive precision and convergence characteristics is provided by the error histograms in Fig. 6a–c. These histograms illustrate the distribution of residual errors across the entire dataset by plotting the frequency of errors within discrete intervals. The resulting distributions exhibit a narrow, symmetric bell-shaped curve centered near zero, indicating that the vast majority of predictions deviate minimally from their target values. The rapid decay of the histogram tails confirms that large errors are infrequent and do not compromise solution quality. Collectively, this strong concentration around zero error demonstrates excellent agreement between the final ANN outputs and the reference computational data, validating the model's accuracy and robust convergence performance. The convergence behavior of the MSE during the training phase is illustrated in Fig. 7a–c. As the principal performance metric, MSE quantifies the average squared deviation between the network's predictions and the target values. The plots track the evolution of this error across the training, testing, and validation phases. The network achieved its optimal performance at distinct epochs for each case specifically at epochs 2000 in each scenario underscoring the robustness and stability of the developed fluid model. Figure 8a–c present a comprehensive fitness analysis of the Error Analysis (EA) solutions across all investigated cases. These graphical representations demonstrate the progressive refinement of function fitness through successive computational stages, revealing how the model iteratively improves its approximation of the underlying physical phenomena. The error plots are constructed for normalized input values spanning from 0 to 1 at fine increments of 0.01, enabling an exceptionally detailed inspection of solution behavior across the entire domain. This high-resolution approach captures subtle variations and localized discrepancies that might otherwise remain undetected with coarser sampling. The resulting fitness profiles confirm that the EA solutions maintain consistent accuracy throughout the parameter space, with error magnitudes remaining uniformly low across all input values, thereby validating the model's reliability and generalization capability. The dynamic learning progression of the artificial neural network under LMA framework is depicted in Fig. 9a–c. These plots trace the progressive refinement of the network's predictions over successive training epochs, revealing a clear evolutionary trajectory from initial coarse approximations toward highly accurate final solutions. This iterative optimization process systematically minimizes prediction errors, enabling the model to simultaneously capture both the global trends and the intricate local details characterizing the coupled velocity, thermal, and solutal fields. By successfully resolving these multi-physics interactions, the network demonstrates its capacity to accurately represent the complex coupling between momentum transport, energy transfer, and mass diffusion mechanisms. The convergence behavior illustrated confirms that the LMA-driven training efficiently navigates the error landscape, achieving stable and precise solutions that faithfully replicate the underlying physical phenomena governing fluid flow on curved stretching surfaces. Finally, the regression analysis is presented in Fig. 10. The correlation coefficients (R) for training, testing, and validation datasets consistently approach unity, indicating an almost perfect linear relationship between predicted outputs and target values. This exceptional correlation across all data subsets provides strong statistical evidence of the proposed LMA model's high predictive accuracy and reliability. The near-unity R-values confirm that the neural network has successfully learned the complex underlying physics governing fluid flow on curved stretching surfaces without over-fitting or under-fitting.

The predictive capability of the selected multi-layer feedforward ANN architecture was compared and contrasted in a detailed manner with two alternative standard regression methodologies: Support Vector Regression (SVR) using Radial Basis Function kernel and ensemble Random Forest (RF) regressor to prove that the chosen multi-layer feedforward ANN architecture has superior predictive capability in this non-Newtonian fluid system. Training and testing splits were produced from a set of base numerical computations and were used in all models. As shown in Table 1, the ANN architecture is a very significant improvement over the other methods, exhibiting a much stronger ability to model the complex, coupled non-linearities of the Williamson–Casson flow field.

**Table 1:** Performance benchmarking of ANN versus alternative models

Predictive target	Performance metric	Alternative SVR model	Alternative RF model	Current proposed ANN
Skin friction $f^{\prime\prime}\left( 0 \right)$	Mean squared error (MSE)	1.24 × 10^–4^	3.85 × 10^–5^	1.12 × 10^–7^
	Coeff. of determination (*R*^*2*^)	0.9854	0.9942	0.9999
Nusselt number $\theta^{\prime}\left( 0 \right)$	MSE	2.15 × 10^–4^	5.12 × 10^–5^	4.38 × 10^–8^
	*R*^*2*^	0.9812	0.9918	1.0000

### Discussion of skin friction, Nusselt number and Sherwood number

**Fig. 11:**
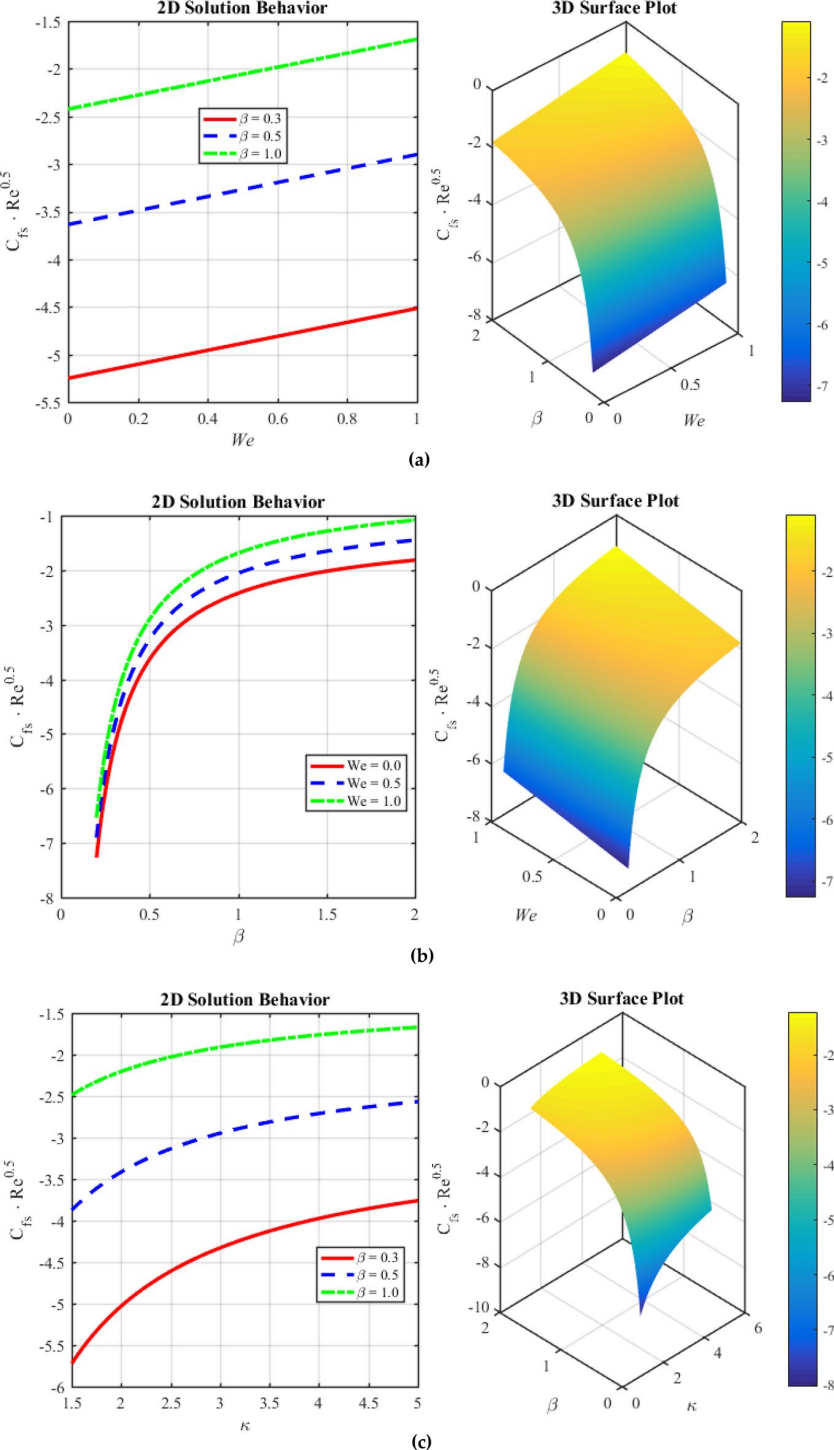
Graphical results for skin friction

**Fig. 12:**
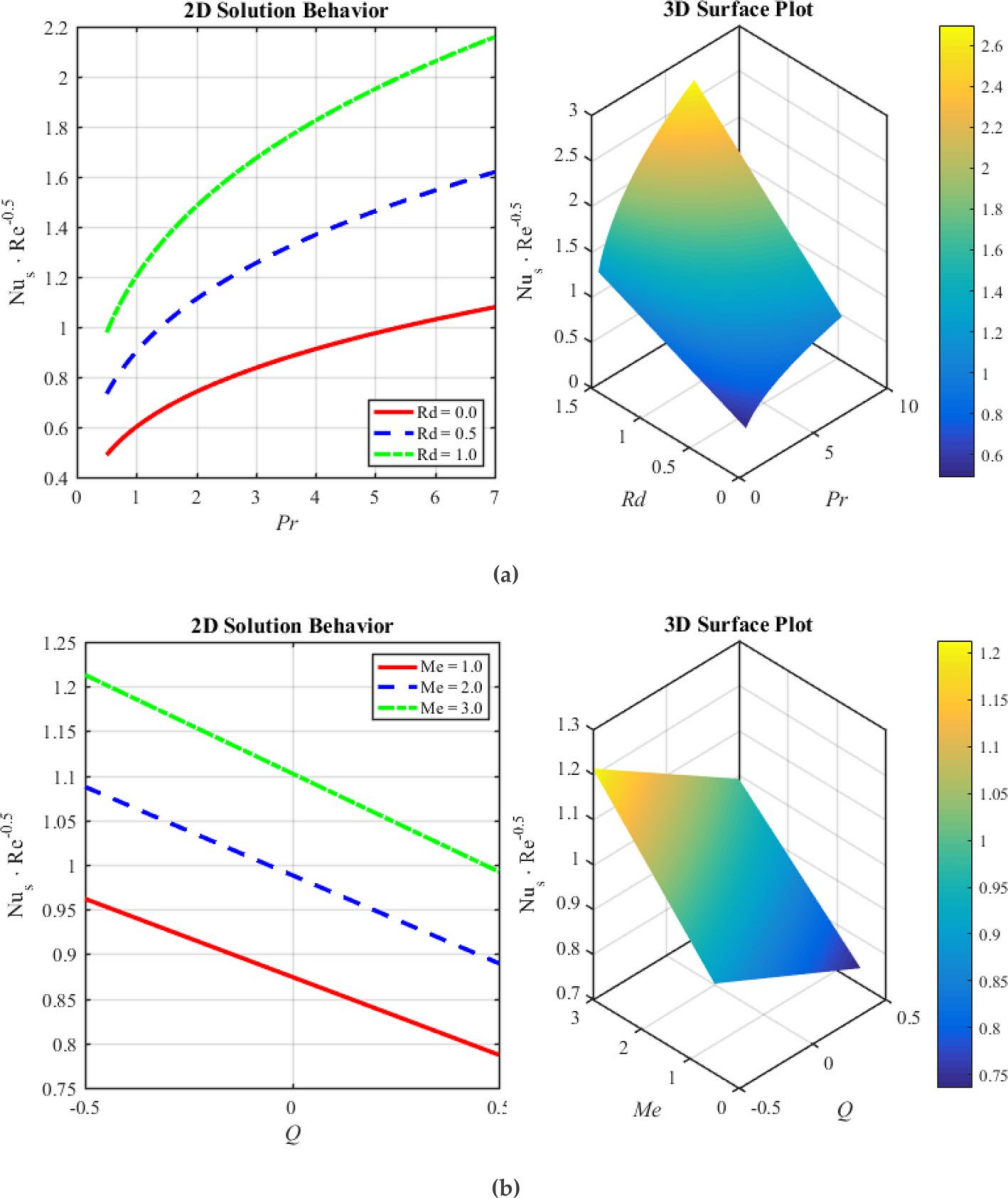
Graphical results for Nusselt number

**Fig. 13:**
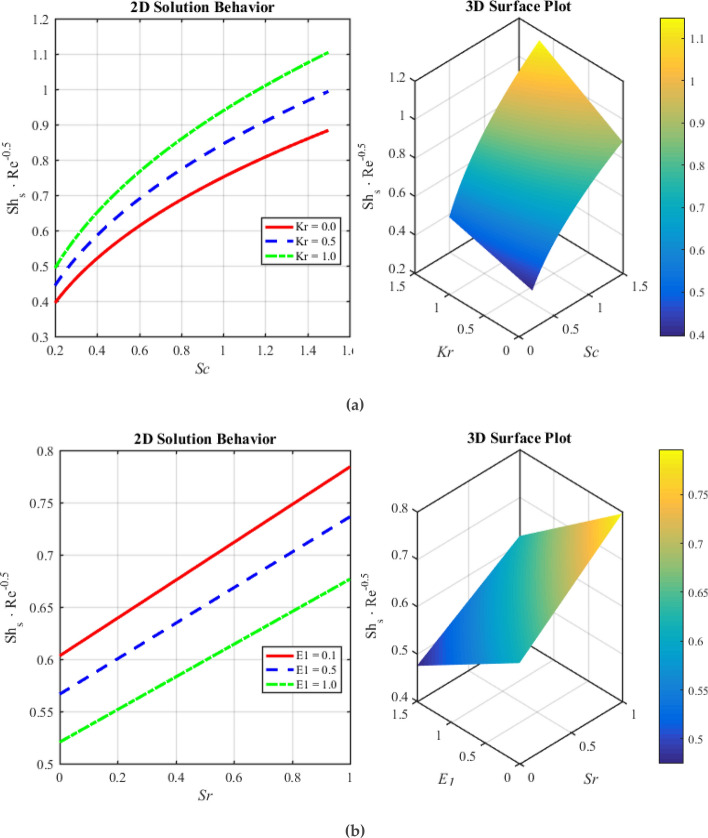
Graphical results for Sherwood number

The effects of various parameters on important quantities are expressed in Figs. 11, 12, 13. Figure 11 a, b express the impression of local Weissenberg number $\left( {We} \right)$ along with Casson factor $\left( \beta \right)$ on skin friction $\left\{ {C_{fs} \left( {Re} \right)^{1/2} } \right\}$. It has deduced from these figures that with a growth in $\left( {We} \right)$ or $\left( \beta \right)$ there is augmentation in $\left\{ {C_{fs} \left( {Re} \right)^{1/2} } \right\}$. Actually an increment in $\left\{ {C_{fs} \left( {Re} \right)^{1/2} } \right\}$ due to the increased $\left( {We} \right)$ or $\left( \beta \right)$ can be explained as when there is an intensification in $\left( \beta \right)$, it implies that the flow will require a higher value of the shear stress for its onset, hence, once the flow starts, it will encounter more resistance as a result of the high plastic viscosity and yield properties of the fluid, thereby enhancing the drag force exerted on the surface. A rise in $\left( {We} \right)$, on the other hand, implies that there is dominance of the elastic forces as opposed to viscous forces, leading to the build-up of energy within the fluid near the surface, this property rises the normal stresses and stretching of polymer molecules. There 3D depictions are also explained in Fig. 11a, b. Figure 11c examines the effect of curvature factor $\left( \kappa \right)$ on the skin friction $\left\{ {C_{fs} \left( {Re} \right)^{1/2} } \right\}$ along with 3D depiction of the effects. A rise in $\left( \kappa \right)$, normally meaning the convex curvature of the curved surface, causes more skin friction owing to the changes brought about to the velocity gradient along the wall. The reason is that an increased curvature results in an enlarged effect on the fluid of the centrifugal and lateral pressure forces. For the case of a convex surface, there is an adverse pressure gradient for the flow that will lead to an increase in the thickness of the boundary layer and hence the velocity shear along the wall. On curved passages increased curvature results in secondary flows, which move the high momentum regions of the flow towards the walls thus steepening the velocity gradient along the wall and hence causing an intensification in skin friction $\left\{ {C_{fs} \left( {Re} \right)^{1/2} } \right\}$. The effect of Prandtl number $\left( {\Pr } \right)$ and heat source parameter $\left( Q \right)$ on Nusselt number $\left\{ {Nu_{s} \left( {Re} \right)^{ - 1/2} } \right\}$ along with 3D depiction of these effects on $\left\{ {Nu_{s} \left( {Re} \right)^{ - 1/2} } \right\}$ are explained in Fig. 12a, b. With a rise in the value of $\left( {\Pr } \right)$, it becomes easier for the fluid to diffuse the momentum rather than heat energy, thereby forming a thinner thermal boundary layer when compared to the velocity boundary layer. The temperature gradient becomes sharper at the wall of curved surface, resulting in better convective heat transfer performance and a greater $\left\{ {Nu_{s} \left( {Re} \right)^{ - 1/2} } \right\}$ as given in Fig. 12a. Figure 12b examines the impression of $\left( Q \right)$ on $\left\{ {Nu_{s} \left( {Re} \right)^{ - 1/2} } \right\}$. Actually, when $\left( Q \right)$ increased, then there is more heat generated internally by the fluid. The result is a rise in temperature of the fluid close to the wall of curved surface, and consequently the temperature gradient also becomes large. As a consequence, there is an intensification in the convective heat transfer coefficient, and hence results a surge in $\left\{ {Nu_{s} \left( {Re} \right)^{ - 1/2} } \right\}$. The effect of Schmidt number $\left( {Sc} \right)$ and Soret number $\left( {Sr} \right)$ on Sherwood number $\left\{ {Sh_{s} \left( {Re} \right)^{ - 1/2} } \right\}$ along with 3D depiction of these effects on $\left\{ {Sh_{s} \left( {Re} \right)^{ - 1/2} } \right\}$ are explained in Fig. 13a, b. A larger $\left( {Sc} \right)$ suggests that the rate of diffusion of momentum is much faster than that of mass diffusion. As such, the concentration boundary layer will be thin as compared to the velocity boundary layer. As a result, the concentration gradient near the wall becomes steeper, thus increasing mass transfer. This means that the profiles of $\left\{ {Sh_{s} \left( {Re} \right)^{ - 1/2} } \right\}$ will ultimately augment as examined in Fig. 13a. The impression of $\left( {Sr} \right)$ on $\left\{ {Sh_{s} \left( {Re} \right)^{ - 1/2} } \right\}$ along with 3D depiction are shown in Fig. 13b. The Soret effect, leads to mass flux because of the temperature gradient present. If the Soret number $\left( {Sr} \right)$ is larger, there is more thermo-diffusion caused by the temperature gradient, which acts against the diffusional flux caused by the concentration difference. This leads to the concentration gradient near the wall becoming smaller, hence reducing Sherwood number $\left\{ {Sh_{s} \left( {Re} \right)^{ - 1/2} } \right\}$.

The effects of various parameters on skin friction $\left\{ {C_{fs} \left( {Re} \right)^{1/2} } \right\}$, Nusselt number $\left\{ {Nu_{s} \left( {Re} \right)^{ - 1/2} } \right\}$ and Sherwood number $\left\{ {Sh_{s} \left( {Re} \right)^{ - 1/2} } \right\}$ are examined in Table 2. It is obvious from this table that with growth in local Weissenberg number $\left( {We} \right)$, Casson factor $\left( \beta \right)$ and curvature factor $\left( \kappa \right)$ there is augmentation in skin friction $\left\{ {C_{fs} \left( {Re} \right)^{1/2} } \right\}$. Intensification in $\left\{ {C_{fs} \left( {Re} \right)^{1/2} } \right\}$ due to escalation in $\left( {We} \right)$, $\left( \beta \right)$, and $\left( \kappa \right)$ is attributed to the fact that fluid elasticity, yield stress, and curvature all contribute to a rise in wall shear stress. With increase in Weissenberg number, there would be increased elastic effects in the fluid, thus leading to higher normal stress and hence increased resistance to deformation by the walls. An increased Casson factor leads to an intensification in yield stress and plastic viscosity, thus requiring additional forces to be exerted to overcome internal fluid resistance. Additionally, increased curvature leads to higher secondary flow rates and centrifugal effects. All these factors work together to create increased intrinsic resistance within the fluid and increased shear rate near the wall, ultimately leading to intensified skin friction $\left\{ {C_{fs} \left( {Re} \right)^{1/2} } \right\}$. With growth in Prandtl number $\left( {\Pr } \right)$ and melting heat parameter $\left( {M_{e} } \right)$ there is augmentation in $\left\{ {Nu_{s} \left( {Re} \right)^{ - 1/2} } \right\}$. The enhancement in $\left\{ {Nu_{s} \left( {Re} \right)^{ - 1/2} } \right\}$ due to rise in $\left( {\Pr } \right)$ and $\left( {M_{e} } \right)$ can be attributed to their combined influence on the thermal boundary layer. An intensification in Prandtl number implies a faster diffusion of momentum compared to thermal energy, resulting in a thin thermal layer at boundary and a steep wall temperature gradient, which rises heat transfer through convection. Moreover, an escalation in melting heat parameter implies the release of more latent heat by the melting, leading to thermal suppression of the boundary layer. The net effect of both factors is a contraction of the thermal boundary layer, making the temperature distribution closer to the wall. The effect is an intensification in heat flux at the wall and, consequently, a rise in Nusselt number. In physical terms, thermal suppression due to poor thermal diffusion capacity of the fluid and melting leads to enhanced heat transfer. With growth in Schmidt number $\left( {Sc} \right)$ and Soret number $\left( {Sr} \right)$ there is augmentation in $\left\{ {Sh_{s} \left( {Re} \right)^{ - 1/2} } \right\}$ while higher factor of activation energy $\left( {E_{1} } \right)$ causes reduction in $\left\{ {Sh_{s} \left( {Re} \right)^{ - 1/2} } \right\}$. Actually, rise in $\left( {Sc} \right)$ makes the concentration boundary layer thinner, increasing the concentration gradient and hence improving the mass transfer process. On the other hand, an intensification in $\left( {Sr} \right)$ (thermophoresis) escalates the mass flux in the direction of the temperature gradient; provided that the thermal conditions are suitable, the second mode of transport process will enhance the Fick’s law of mass transport, thus increasing the Sherwood number $\left\{ {Sh_{s} \left( {Re} \right)^{ - 1/2} } \right\}$. But when the activation energy is increased, then $\left\{ {Sh_{s} \left( {Re} \right)^{ - 1/2} } \right\}$ is decreased due to change in the properties of the fluid caused by the temperature-dependent reaction rate.

**Table 2:** Numerical results for skin friction, Nusselt number and Sherwood number

We	$\beta$	$\kappa$	*Pr*	$M_{e}$	*Sc*	$E_{1}$	*Sr*	$f^{\prime\prime}\left( 0 \right)$	$\theta^{\prime}\left( 0 \right)$	$\varphi^{\prime}\left( 0 \right)$
0.0	0.3	1.5	6.0	1.0	1.0	1.5	2.0	− 3.634800		
0.5								− 3.267806		
1.0								− 2.900813		
	0.3							− 5.030070		
	0.6							− 3.414604		
	0.9							− 2.203004		
		1.5						− 3.873210		
		3.0						− 2.946737		
		4.5						− 2.565775		
			6.0						0.543200	
			7.0						0.814800	
			8.0						1.086400	
				1.0					0.874552	
				2.0					0.988624	
				3.0					1.102696	
					1.0					0.414358
					3.0					0.643020
					5.0					0.848470
						1.5				0.603214
						2.0				0.566470
						2.5				0.520540
							2.0			0.583464
							3.0			0.634446
							4.0			0.702423

**Fig. 14:**
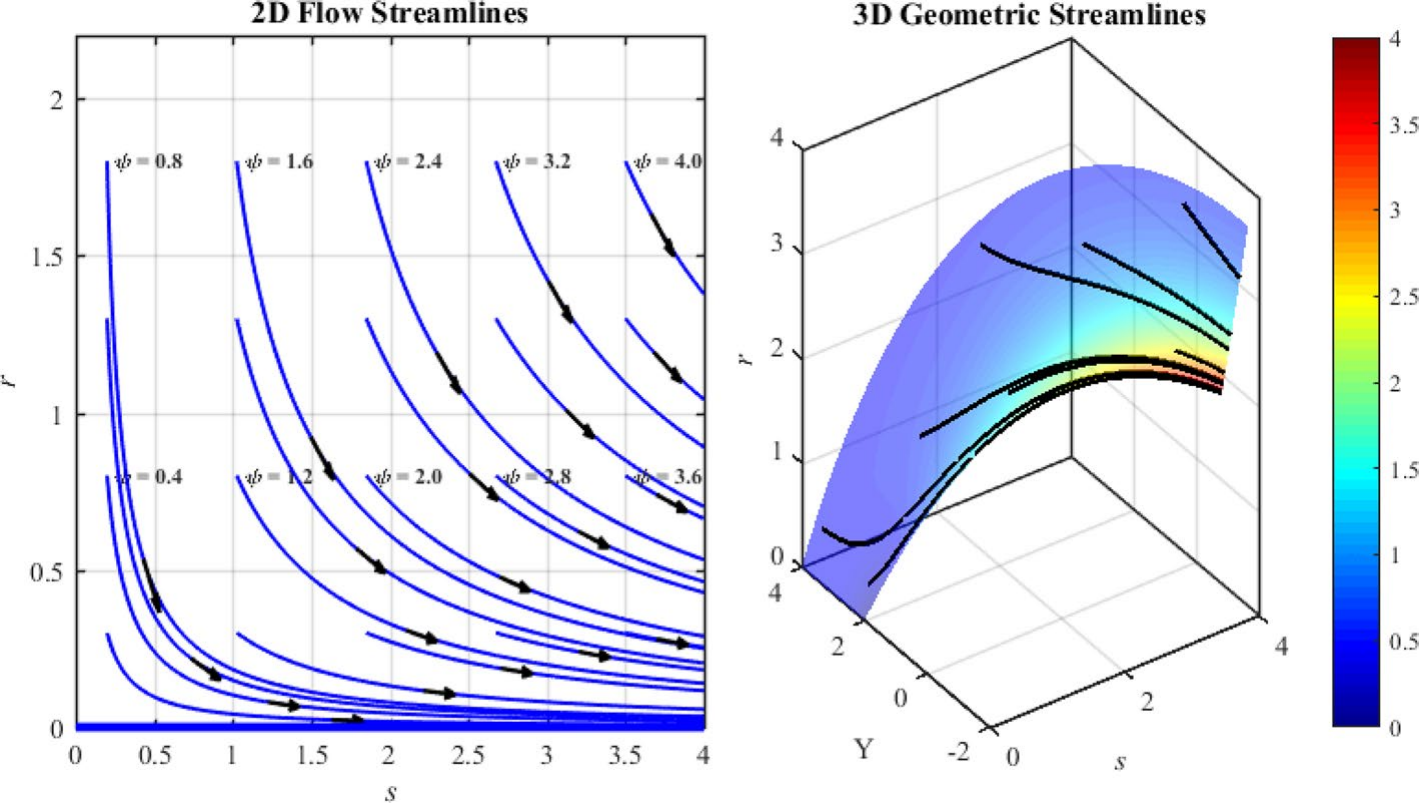
Streamlines graph for the present model

Two-dimensional (2D) and three-dimensional (3D) streamline tracking is used to model and evaluate the visual aspects of the fluid flow trajectory and the growth of the boundary layer over the slippery curved expanding sheet, as shown in the Fig. 14. It is clearly noted that the value of the stream function increases systematically from ψ = 0.4 near the stretching interface to ψ = 4.0 in the upper cross-stream domain. The streamlines near the solid boundary (*r* = 0) curve severely and turn parallel to the horizontal axis, making it very clear that the physical drag minimization and velocity slip effects along the solid boundary are the dominant effects for the WC fluid continuum. The right side complements this representation, by plotting the same flow paths, directly onto the quarter-cylinder ‘3D’ geometric surface layout. The trajectory tracks of the stream on the 3D surface are exactly the same as those in the 2D stream, and there is a consistency of structures throughout the domain, with the spatial coordinate transformation valid. In addition, the 3D color gradient map shows the variations of the velocity field intensity, with the maximum velocity adjacent to the leading expanding zone and gradually decreasing to the undisturbed ambient free-stream matrix. It's a very strict visual comparison between the 2D tracks and the 3D block surface, which is very helpful to check the mathematical fidelity of our scheme and to understand the entrainment mechanism in the flow over a curved material processing channel.

**Fig. 15:**
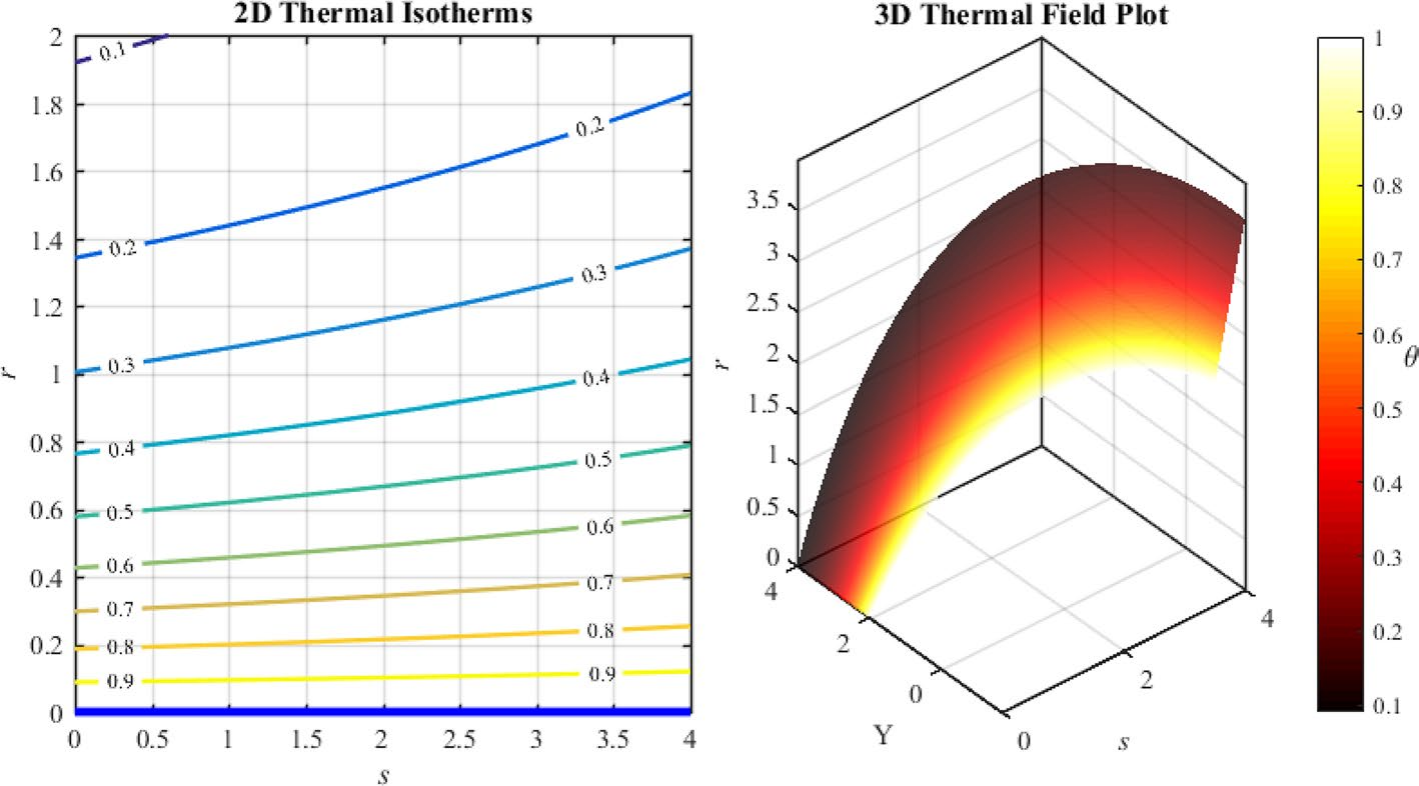
2D thermal isotherms and 3D thermal field distribution over a curved stretching sheet

The effects of melting heat transfer and radiative heat flux are examined in a systematic and comprehensive manner for the thermal distribution and boundary layer development under the collective influence of these two effects, using 2D and 3D isothermal plot as shown in the Fig. 15. The left-hand side shows 2D thermal isotherms plotted. Near the solid curved surface (*r* = 0), the isotherms are packed close together with high temperature values ($\theta$ = 0.9) which physically represents the high thermal gradients created by the expanding surface. These temperature profiles tend to converge toward the ambient free stream state ($\theta$ → 0.1) as the normal radial distance r gets larger. Most importantly, the isotherms are curved upward and increase in length in the streamwise direction (*s*) which is a visual testament to the growth and expansion of the thermal boundary layer as the fluid flows downstream along the curved sheet.

#### Results validation

A comprehensive validity is carried out to ensure accuracy and reliability of the present numerical framework. The present results of the energy transfer rate $\theta^{\prime}\left( 0 \right)$ are compared with the benchmark results of Khan et al. [55] for different values of the relevant physical parameters. Comparing the presently reported numerical results with the previous published results (as summarized in Table 3), it can be noticed that an outstanding level of agreement exists for all test cases. For instance, at baseline values ($\varepsilon$ = 0.4, $\beta_{m}$ = 0.3, *Pr* = 6.8, and *Rd* = 0.4), the current scheme yields $\theta^{\prime}\left( 0 \right)$ = 0.3310767), which is closely related to 0.33107 obtained by Khan et al. [55]. For this very high precision and very small residual difference, all over the whole parametric range, it is a very strong confirmation of the robustness and fidelity of the solution procedure bvp4c. Therefore, the validated numerical model can be considered as a very reliable structure for creating the data arrays used in the following trained artificial neural network (ANN) structure.

**Table 3:** Numerical results validation versus the published study

$\varepsilon$	$\beta_{m}$	*Pr*	*Rd*	$\varepsilon$	***Ec***	Khan et al. [55] $\theta^{\prime}\left( 0 \right)$	Present results $\theta^{\prime}\left( 0 \right)$
0.4	0.3	6.8	0.4	0.1	0.1	0.33107	0.3310767
0.7						0.33097	0.3309798
1.0						0.33002	0.3300234
	0.7					0.33224	0.3322409
	1.1					0.33215	0.3321525
	1.5					0.33207	0.3320774
		6.9				0.33382	0.3338286
		10.0				0.33531	0.3353135
		10.11				0.33680	0.3368027
			0.6			0.37926	0.3792685
			0.8			0.42606	0.4260638
			1.0			0.47274	0.4727436
				0.4		0.33261	0.3326128
				0.7		0.33289	0.3328958
				1.0		0.33317	0.3331746
					0.4	0.33473	0.3347378
					0.7	0.33714	0.3371446
					1.0	0.33955	0.3395525

## Conclusions

The current work systematically examines the Williamson-Casson nanofluid flow over a slippery curved expanding surface while simultaneously incorporating the Cattaneo-Christov heat flux model. The flow is affected by melting heat transfer conditions at boundary, activation energy, chemical reactivity and Soret/Dufour effects. The modeled equations have solved through bvp4c approach in dimensionless form. The solution obtained from this approach is then used to provide a dataset for Artificial Neural Network (ANN) approach. After detailed examination of the work, it has revealed that:

- Velocity profiles augmented with growth in thermal buoyancy factor and velocity slip parameter while declined with augmentation in magnetic parameter, ferrohydrodynamic interaction and Weissenberg number.
- Thermal profiles escalated with augmentation in heat source/sink parameter, heat dissipation factor, unsteadiness factor, radiation factor and Dufour number while declining with augmentation in melting heat parameter, thermal buoyancy factor and thermal relaxation factor.
- Concentration profiles augmented with growth in Soret number and activation energy while declining with escalation in Schmidt number and chemical reaction rate.
- The analysis of the histograms across the entire dataset by plotting the frequency of errors within discrete intervals confirms the validity and reliability of the model.
- Visual assessment of model precision and convergence through residual error distribution have been explained through error histograms**.**
- The effects of various factors on drag force and Nusselt number have been examined graphically.

### Limitations and future directions

The present study is able to provide a high fidelity numerical and machine-learning tool for the study of non-Newtonian transport phenomena, but it is important to note that some limitations exist. This study is physically based on a steady state or simplified unsteady continuous phase domain, and assumes the thermal conductivity to be a constant factor. Moreover, the fluid–solid interaction is restricted to two-dimensional profiles of boundary layers over smooth curved geometries without accounting for surface roughness and/or 3D rotational effects.

To rise the above limitations, future research directions will be directed towards extension of this model for the study of three-dimensional swirling flows over rough and textured curved stretching surfaces. In addition, the model including variable thermophysical properties such as variable thermal conductivity and dynamic viscosity will improve the accuracy of the model under extreme thermal gradient conditions. Finally, future computational studies will focus on using a deeper physics informed neural network (PINN) to directly solve the governing differential equations, bypassing the use of conventional numerical solution methods, and allowing for real-time engineering optimizations.

## Data Availability

The data that support the findings of this study are available from the corresponding author upon reasonable request.

## List of symbols

$$t$$: Time (s)
$$u_{w}$$: Stretching velocity (m/s
$$\left( {u,v} \right)$$: Velocity component
$$\rho$$: Density (kg/m^3^)
${\Gamma }$: Material time constant
$$\mu_{0}$$: Magnetic permeability
$\beta_{T}$: Coefficient of thermal expansion
$$H$$: Magnetic field
$$Q_{0}$$: Heat source
$$\beta_{0}$$: Magnetic field
$q_{r}$: Radiative heat flux
$$\alpha$$: Thermal diffusivity (m^2^/s)
$D_{T}$: Thermophoretic diffusion term
$$B_{m}$$: Magnetic dipole
$$T_{\infty }$$: Ambient temperature
$$\beta_{m}$$: Ferrohydrodynamic interaction
*Sr*: Soret number
*Gr*: Local Grashof number
$$M^{*}$$: Magnetic parameter
$$\varepsilon$$: Curie temperature
$C_{H}$: Thermal relaxation parameter
CCHF: Cattaneo-Christov heat flux
ANN: Artificial Neural Network
$$a$$: Stretching rate
$\alpha^{*}$: Reciprocal to the dimension of time
*R*: Radius (m)
$$\upsilon$$: Kinematic viscosity (m^2^/s)
$$p$$: Pressure
$$\beta$$: Casson factor
$$g$$: Gravitational acceleration
$$M$$: Magnetization
$$\sigma$$: Electrical conductivity (S/m)
$C_{p}$: Specific heat (J/kg·K)
*T*: Temperature
$\tau$: Ratio of effective heat capacity
$\lambda_{1}$: Thermal relaxation time
$$L_{s}$$: Slip velocity
$$T_{w}$$: Surface temperature
*k*: Thermal conductivity (W/m·K)
$$\delta^{*}$$: Unsteadiness parameter
*b*: Dimensionless distance
$K_{r}$: Rate of chemical reaction
$\lambda_{T}$: Thermal buoyancy parameter
*Rd*: Thermal radiation parameter
*We*: Local Weissenberg number
WC: Williamson-Casson
MHD: Magnetohydrodynamic
